# The Strongly Asynchronous Massive Access Channel

**DOI:** 10.3390/e25010065

**Published:** 2022-12-29

**Authors:** Sara Shahi, Daniela Tuninetti, Natasha Devroye

**Affiliations:** Electrical and Computer Engineering Department, University of Illinois at Chicago, Chicago, IL 60607, USA

**Keywords:** multiple access channels, massive access, asynchronous communication

## Abstract

This paper considers the Strongly Asynchronous, Slotted, Discrete Memoryless, Massive Access Channel (SAS-DM-MAC) in which the number of users, the number of messages, and the asynchronous window length grow exponentially with the coding blocklength with their respective exponents. A joint probability of error is enforced, ensuring that all the users’ identities and messages are correctly identified and decoded. Achievability bounds are derived for the case that different users have similar channels, the case that users’ channels can be chosen from a set which has polynomially many elements in the blocklength, and the case with no restriction on the users’ channels. A general converse bound on the capacity region and a converse bound on the maximum growth rate of the number of users are derived. It is shown that reliable transmission with an exponential number of users with an exponential asynchronous exponent with joint error probability is possible at strictly positive rates.

## 1. Introduction

One of the main attributes of the Internet of the Things (IoT) paradigm is the wireless connectivity of large a number of distributed and uncoordinated devices. Each device is automated, and is allowed to transmit random bursts of data without network or human coordination. A contention-based/grant-based network access requires multiple rounds of back and forth transmissions between the device and the network to acquire synchronization, identify users, and grant them allocations. This signaling overhead and power consumption is not justified for power-limited devices with sporadic traffic patterns and motivates a grant-free network access implementation. An un-coordinated network access leads to a new traffic patterns and collision resolutions over the wireless network. A foundational study of such a system is needed.

We propose a novel communication and multiple-access model that captures these new network characteristics. The formal model definition is given in Section 2, but in order to demonstrate the differences between our proposed model and existing work in the literature, we briefly introduce our model parameters here for the Strongly Asynchronous Slotted Discrete Memoryless Massive Access Channel (SAS-DM-MAC). In a SAS-DM-MAC, the number of users $K_{n}:=e^{n\nu }$ increases exponentially with blocklength *n* with *occupancy* exponent ν≥0. Moreover, the users are strongly asynchronous, meaning, they transmit at a randomly chosen time slot within a window of length $A_{n}:=e^{n\alpha }$ slots, each slot of length *n*, where α≥0 is the *asynchronous* exponent. In addition, when active, each user chooses uniformly at random one message to transmit from a set of $M_{n}:=e^{nR}$ messages, where R≥0 is the transmission *rate*. All transmissions are sent to an access point and the receiver is required to *jointly decode and identify all users*. The goal is to characterize the set of all achievable (R,α,ν) triplets.

### 1.1. Past Work

Strongly asynchronous communication was first introduced in [1] for synchronization of a single user, and later extended in [2] for synchronization with positive transmission rate.

In [3] the authors of [2] made a brief remark about a “multiple access collision channel” extension of their original single-user model. In this model, any collision of users (i.e., users who happen to transmit in the same block) is assumed to result in output symbols that appear distributed as noise. The error metric is taken to be the *per user* probability of error, which is required to be vanishing for all but a vanishing fraction of users. In this scenario, it is fairly easy to quantify the capacity region for the case that the number of users are less than the square root of the asynchronous window length (i.e., in our notation ν<α/2). However, finding the capacity of the “multiple access collision channel” for *joint* probability of error, as opposed to per user probability of error, is much more complicated and requires novel achievability schemes and novel analysis tools. This is the main subject and contribution of this paper.

Recently, motivated by the emerging machine-to-machine type communications and sensor networks, a large body of work has studied “many-user” versions of classical multiuser channels as pioneered in [4]. In [4] the number of users is allowed to grow at most linearly with the blocklength *n*. A full characterization of the capacity of the synchronous Gaussian (random) many access channel was given [4]. In [5], the author studied the synchronous massive random access channel where the total number of users increases linearly with the blocklength *n*. However, the users are restricted to use the same codebook and only a per user probability of error is enforced. In the model proposed here, the users are strongly asynchronous, the number of users grows exponentially with the blocklength, and we enforce a joint probability of error over all users.

In [6,7] a broadcast channel model was introduced where each receiver is only interested in decoding its intended messaged (and not all messages). For a per user/per pair of users probability of error criteria doubly exponential number of receivers was shown to be identifiable by using a randomized code. In this work we study a multi-access channel model with non-cooperative transmitters and a single receiver which needs to decode all messages from all users.

Training based synchronization schemes (i.e., pilot signals) was proven to be suboptimal for bursty communications in [2]. Rather, one can utilize the users’ statistics at the receiver for synchronization or user identification purposes. The identification problem (defined in [8]) is a classical problem considered in hypothesis testing. In this problem, a finite number of distinct sources each generates a sequence of i.i.d. samples. The problem is to find the underlying distribution of each sample sequence, given the constraint that each sequence is generated by a distinct distribution.

All studies on identification problems assume a fixed number of sequences. In [9], authors study the Logarithmically Asymptotically Optimal (LAO) Testing of identification problem for a finite number of distributions. The authors in [10] also propose a test for a generalized Neyman-Pearson-like optimality criterion to match multiple yet finite number of sequences to their source distributions.

In contrast, we allow the number of users to increase exponentially with the blocklength. We assume that the users are strongly asynchronous and may transmit randomly anytime within a time window that is exponentially large in the blocklength. We require the receiver to recover both the transmitted messages and the users’ identities under a joint probability of error criteria. By allowing the number of sequences to grow exponentially with the number of samples, the number of different possibilities (or hypotheses) would be doubly exponential in the blocklength and the analysis of the optimal decoder becomes much more challenging than classical (with constant number of distributions) identification problems. These differences in modeling the channel require a number of novel analytical tools.

### 1.2. Contribution

We consider (R,α,ν), the capacity region of the SAS-DM-MAC, where we require the *joint* probability of error to vanish. Our contributions are as follows:

- We propose a new achievability scheme that supports strictly positive values of (R,α,ν) for identical channels for the users.
- We define a new massive identification paradigm in which we allow the number of sequences in a classical identification problem to increase exponentially with the sequence blocklength (or sample size). We find asymptotically matching upper and lower bounds on the probability of identification error for this problem. We use this result in our SAS-DM-MAC model to recover the identity of the users.
- We propose a new achievability scheme for the case that the channels are chosen from a set of conditional distributions. The size of the set increases polynomially in the blocklength *n*. In this case, the channel statistics themselves can be used for user identification.
- We propose a new achievability scheme without imposing any restrictive assumptions on the users’ channels. We show that strictly positive (R,α,ν) are possible.
- We propose a novel converse bound for the capacity of the SAS-DM-MAC.
- We show that for ν>α, not even reliable synchronization is possible.

These results were presented in parts in [11,12,13].

### 1.3. Paper Organization

In Section 2 we introduce the notation and problem formulation for SAS-DM-MAC and the identification problem. We present our main results in Section 3. Section 4 concludes the paper with a discussion and future work. Proofs are provided in the Appendix.

## 2. Notations and Problem Formulation

We first introduce the notation used in the SAS-DM-MAC and then formally define the problem.

### 2.1. Notation

Capital letters represent random variables that take on lower case letter values in calligraphic letter alphabets. The notation $a_{n}≐e^{nb}$ means $\lim_{n\to \infty }\frac{\log a_{n}}{n}=b$. We write [M:N], where M,N∈Z,M≤N, to denote the set {M,M+1,…,N}, and [K]:=[1:K]. $y^{n}$ is used to denote $y^{n}:=\left[y_{1},\ldots ,y_{n}\right]$. The *n*-fold cartesian product of a set *S* is denoted by $S^{n}$.

A transition probability/channel from X to Y is denoted by Q(y|x) (or *Q* by short), ∀(x,y)∈X×Y, and the output marginal distribution induced by $P\in P_{X}$ through the channel *Q* as

(1) $$\begin{array}{c} \left[PQ\right]\left(y\right):=\sum_{x\in X}P\left(x\right)Q\left(y|x\right),\forall y\in Y, \end{array}$$

where $P_{X}$ is the space of all distributions on X. We define the shorthand notation

(2) $$\begin{array}{cc} Q_{x}\left(y\right) & :=Q(y|x),\forall y\in Y. \end{array}$$

For a MAC channel $Q\left(y|x_{1},\ldots ,x_{K}\right),\forall \left(x_{1},\ldots ,x_{K},y\right)\in X_{1}\times \ldots \times X_{K}\times Y$, we define the shorthand notation

(3) $$\begin{array}{cc} Q_{S}\left(y|x_{S}\right) & :=Q\left(y|x_{S},{⋆}_{S^{c}}\right),\forall S\subseteq \left[K\right], \end{array}$$

to indicate that users indexed by *S* transmit $x_{i}\in X_{i},\forall i\in S$, and users indexed by $S^{c}=\left[K\right]\setminus S$ transmit their respective idle symbol ${⋆}_{j}\in X_{j}\;\forall j\in S^{c}$. When |S|=1 and |S|=0, we respectively use

(4) $$\begin{array}{cc} Q_{i}\left(y|x_{i}\right) & :=Q_{\left\{i\right\}}\left(y|x_{i}\right)=Q\left(y|{⋆}_{1},\ldots ,{⋆}_{i-1},x_{i},{⋆}_{i+1},\ldots ,{⋆}_{K}\right),\\ Q_{⋆}\left(y\right) & :=Q_{\left\{\emptyset \right\}}\left(y|x_{i}\right)=Q\left(y|{⋆}_{1},\ldots {⋆}_{K}\right). \end{array}$$

The *P*-type set and the *V*-shell of the sequence $x^{n}$ are defined, respectively, as

(5) $$\begin{array}{cc} T(P) & :=\left\{x^{n}:\frac{N(a|x^{n})}{n}=P\left(a\right),\forall a\in X\right\}, \end{array}$$

(6) $$\begin{array}{cc} T_{V}\left(x^{n}\right) & :=\left\{y^{n}\;:\;\frac{N\left(a,b|x^{n},y^{n}\right)}{N(a|x^{n})}=V\left(b|a\right),\forall \left(a,b\right)\in \left(X,Y\right)\right\}, \end{array}$$

where $N\left(a,b|x^{n},y^{n}\right)=\sum_{i=1}^{n}1_{\left\{\begin{array}{c} x_{i}=a\\ y_{i}=b \end{array}\right\}}$ is the number of joint occurrences of (a,b) in the pair of sequences $\left(x^{n},y^{n}\right)$ and where $N(a|x^{n})$ denotes the number of occurrences of a∈X in the sequence $x^{n}$. The empirical distribution of the sequence $x^{n}$ is

(7) $$\begin{array}{c} {\hat{P}}_{x^{n}}\left(a\right):=\frac{1}{n}N\left(a|x^{n}\right)=\frac{1}{n}\sum_{i=1}^{n}1_{\left\{x_{i}=a\right\}},\forall a\in X. \end{array}$$

If in using (7) the target sequence $x^{n}$ is clear from the context, we drop the subscript $x^{n}$ in ${\hat{P}}_{x^{n}}\left(\cdot \right)$.

We also use the notation $X^{n}\overset{i.i.d}{∼}P$ when all random variables in the random vector $X^{n}$ are generated i.i.d according to distribution *P*.

We use $D(P_{1}\|P_{2})$ to denote the *Kullback Leibler divergence* between distribution $P_{1}$ and $P_{2}$, and

(8) $$\begin{array}{c} D(Q_{1}\|Q_{2}\left|P\right):=\sum_{\left(x,y)\in (X\times Y\right)}P\left(x\right)Q_{1}\left(y|x\right)\log\frac{Q_{1}\left(y|x\right)}{Q_{2}\left(y|x\right)} \end{array}$$

for the conditional Kullback Leibler divergence. We let

(9) $$\begin{array}{c} I(P,Q)=D(Q\|[PQ]\;|\;P) \end{array}$$

denote the *mutual information* between random variables *X* and *Y* jointly distributed according to $P_{X,Y}\left(x,y\right)=P\left(x\right)Q\left(y|x\right)$. As for binary functions, we use

$$\begin{array}{cc} d(p\|q) & :=p\log\frac{p}{q}+\left(1-p\right)\log\frac{1-p}{1-q},\\ p*q & :=p(1-q)+(1-p)q,\\ h(p) & :=-p\log(p)-(1-p)\log(1-p) \end{array}$$

to represent binary divergence, convolution and entropy functions, respectively.

The *Chernoff distance* and the *Bhatcharrya distance* between two distributions $P_{1},P_{2}\in P_{X}$ are respectively defined as

(10) $$\begin{array}{cc} C(P_{1},P_{2}) & :=\underset{0\leq t\leq 1}{\sup}-\log\left(\sum_{x\in X}P_{1}{\left(x\right)}^{t}P_{2}{\left(x\right)}^{1-t}\right), \end{array}$$

(11) $$\begin{array}{cc} B(P_{1},P_{2}) & :=-\log\left(\sum_{x\in X}\sqrt{P_{1}\left(x\right)P_{2}\left(x\right)}\right). \end{array}$$

We extend this definition and introduce the quantities

(12a) $$\begin{array}{cc} C(P_{i},Q_{i},P_{j},Q_{j}) & :=\underset{0\leq t\leq 1}{\sup}\mu_{i,j}\left(t\right), \end{array}$$

(12b) $$\begin{array}{cc} \mu_{i,j}\left(t\right) & :=-\log\sum_{x_{i}\in X,x_{j}\in X,y\in Y}P_{i}\left(x_{i}\right)P_{j}\left(x_{j}\right)Q_{i}{\left(y|x_{i}\right)}^{1-t}Q_{j}{\left(y|x_{j}\right)}^{t}, \end{array}$$

$$\begin{array}{cc} C(.,Q_{⋆},P_{j},Q_{j}) & :=\underset{0\leq t\leq 1}{\sup}-\log\left(\sum_{x\in X,y\in Y}P_{j}\left(x\right)Q_{⋆}{\left(y\right)}^{1-t}Q_{j}{\left(y|x\right)}^{t}\right). \end{array}$$

We also use

(13) $$\begin{array}{cc} E_{r}\left(R,P,Q\right) & :=\max_{0\leq \rho \leq 1}\left[E_{0}\left(R,P,Q,\rho \right)-\rho R\right], \end{array}$$

(14) $$\begin{array}{cc} E_{sp}\left(R,P,Q\right) & :=\underset{\rho >0}{\sup}\left[E_{0}\left(R,P,Q,\rho \right)-\rho R\right] \end{array}$$

to denote the *random coding* and the *sphere packing* error exponent of channel Q(y|x), with input distribution P(x) and rate *R* as defined in [14] where

(15) $$\begin{array}{c} E_{0}\left(R,P,Q,\rho \right):=-\log\sum_{y\in Y}{\left(\sum_{x\in X}P\left(x\right)Q{\left(y|x\right)}^{\frac{1}{1+\rho }}\right)}^{1+\rho }. \end{array}$$

### 2.2. SAS-DM-MAC Problem Formulation

Let *M* be the number of messages (the same for each user), *A* be the number of blocks, and *K* be the number of users.

An (M,A,K,n,ϵ) code for the SAS-DM-MAC consists of:

- A message set [M], for each user i∈[K].
- An encoding function $f_{i}:\left[M\right]\to X^{n}$, for each user i∈[K]. We define
  (16) $$\begin{array}{c} x_{i}^{n}\left(m\right):=f_{i}\left(m\right). \end{array}$$
- Each user i∈[K] chooses a message $m_{i}\in \left[M\right]$ and a block index $t_{i}\in \left[A\right]$ (called ‘active’ block henceforth), both uniformly at random. It then transmits $\left[{⋆}_{i}^{n(t_{i}-1)}\;x_{i}^{n}\left(m_{i}\right)\;{⋆}_{i}^{n(A-t_{i})}\right]$, where ${⋆}_{i}\in X$ is the designated idle symbol for user *i*.
- A destination decoding function
  (17) $$\begin{array}{c} g:Y^{nA}\to {\left([A]\times [M]\right)}^{K} \end{array}$$
  such that its associated probability of error, $P_{e}^{\left(n\right)}$, satisfies $P_{e}^{\left(n\right)}\leq \epsilon$ where
  (18) $$\begin{array}{c} P_{e}^{\left(n\right)}:=\frac{1}{{\left(AM\right)}^{K}}\sum_{\left(t_{1},m_{1}\right),\ldots ,\left(t_{K},m_{K}\right)}P\left[g\left(Y^{nA}\right)\neq \left(\left(t_{1},m_{1}\right),\ldots ,\left(t_{K},m_{K}\right)\right)|H_{\left(\left(t_{1},m_{1}\right),\ldots ,\left(t_{K},m_{K}\right)\right)}\right], \end{array}$$
  where the hypothesis that user i∈[K] has chosen to transmit message $m_{i}\in \left[M\right]$ in block $t_{i}\in \left[A\right]$ is denoted by $H_{\left(\left(t_{1},m_{1}\right),\ldots ,\left(t_{K},m_{K}\right)\right)}$.

A tuple (R,α,ν) is said to be *achievable* if there exists a sequence of codes $\left(e^{nR},e^{n\alpha },e^{n\nu },n,\epsilon_{n}\right)$ with $\lim_{n\to \infty }\epsilon_{n}=0$. The capacity region of the SAS-DM-MAC at asynchronous exponent α, occupancy exponent ν, and rate *R*, is the closure of all possible achievable (R,α,ν) triplets.

**Remark** **1.** *We should emphasize that we are focusing our attention on discrete memoryless channels (DMC) where a user not transmitting/being idle is equivalent to sending that user’s idle symbol in the DMC model, just as, in a Gaussian channel, a user not sending corresponds to sending the symbol ‘0’. We do not study Continuous channels in this work.*

**Figure 1:**
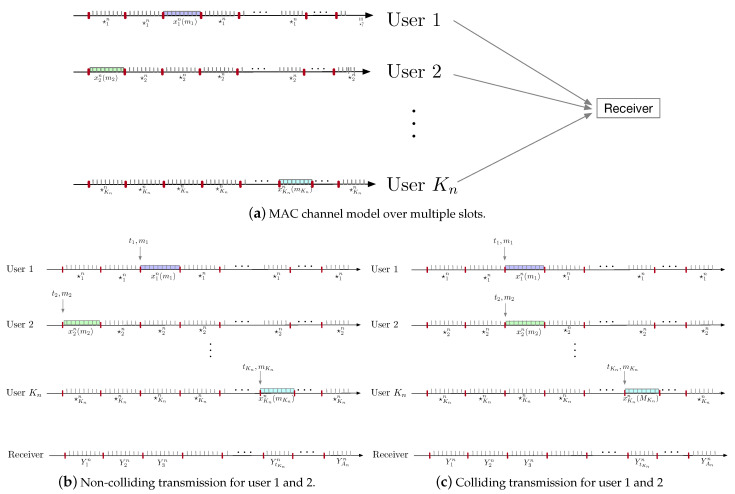
Transmitted sequence for each user and the received sequence in colliding and non-colliding transmission time for user 1 and 2.

A depiction of a timeline for our channel model is provided in Figure 1a and for specific cases: one in which user 1 and 2 are not simultaneously transmitting (Figure 1b) and another in which user 1 and 2 are simultaneously transmitting (Figure 1c).

### 2.3. Identification Problem Formulation

We first summarize the notation specifically used in this Section (and in Appendix B) in Table 1 and then introduce the problem formulation.

Assume $P:=\left\{P_{1},\ldots ,P_{T_{n}}\right\}\subseteq P_{X}$ consist of $T_{n}$ distinct distributions on X and let random variable Σ be uniformly distributed over $S_{T_{n}}$ (see definition in Table 1). Let $\sigma \in S_{T_{n}}$ and let $x^{nT_{n}}=\left[x_{1}^{n},\ldots ,x_{T_{n}}^{n}\right]$ be a sample vector of $X^{nT_{n}}=\left[X_{1}^{n},\ldots ,X_{T_{n}}^{n}\right]$, in which $X_{i}^{n}\overset{i.i.d}{∼}P_{\sigma_{i}}^{n},\forall i\in \left[T_{n}\right]$. Given $x^{nT_{n}}$, we would like to find the permutation σ. More specifically, we are interested in finding a permutation $\hat{\sigma }:X^{nT_{n}}\to S_{T_{n}}$ in which $X_{i}^{n}\overset{i.i.d}{∼}P_{{\hat{\sigma }}_{i}},\;\forall i\in \left[T_{n}\right]$. Let $\hat{\Sigma }=\hat{\sigma }\left(X^{nT_{n}}\right)$.

The average probability of error for the set of distributions *P* is defined by

(19) $$\begin{array}{cc} P_{e}^{\left(n\right)} & =P\left[\hat{\Sigma }\neq \Sigma \right]=\frac{1}{(T_{n})!}\sum_{\sigma \in S_{T_{n}}}P\left[\hat{\Sigma }\neq \sigma \right], \end{array}$$

where in (19) the measure P over $\left(X_{1}^{n},...,X_{T_{n}}^{n}\right)$ is $X_{i}^{n}\overset{i.i.d}{∼}P_{\sigma_{i}},\forall i\in \left[T_{n}\right]$.

We say that a set of distributions *P* is *identifiable* if $\lim_{n\to \infty }P_{e}^{\left(n\right)}\to 0$.

## 3. Main Results

We first introduce an achievable region for the case that different users have identical channels (in Theorem 1). We then derive the identification criteria (in Theorem 2) and use it in the study of a more general case of massive communications in which the users’ channels belong to a set of conditional probability distributions of polynomial size in *n* (in Theorem 3). In this case, we use the output statistics to distinguish and identify the users. Afterwards, we remove all conditions on the users’ channels and derive an achievability bound on the capacity of the SAS-DM-MAC in (Theorem 4). We also propose a converse bound on the capacity of general SAS-DM-MAC (in Theorem 5) and a converse bound on the number of users (in Theorem 6).

### 3.1. Users with Identical Channels

The following theorem is an achievable region for the SAS-DM-MAC for the case that different users have identical channels toward the base station when they are the sole active user.

**Theorem** **1.** *For a SAS-DM-MAC with $Q_{\left\{i\right\}}\left(y|x\right)=W\left(y|x\right)$ (recall Definition (4)) for all users, the following (R,α,ν) region is achievable*

(20) $$\begin{array}{c} \underset{\begin{array}{c} P\in P_{X}\\ \lambda \in [0,1] \end{array}}{⋃}\left\{\begin{array}{cc} \nu & <\frac{\alpha }{2}\\ \nu & <\min\left\{D(W_{\lambda }\|W|P),E_{r}\left(R+\nu ,P,W\right)\right\}\\ \alpha +R+\nu & <D(W_{\lambda }\|Q_{⋆}|P)\\ R+\nu & <I(P,W) \end{array}\right\}, \end{array}$$

*where*

(21) $$\begin{array}{c} W_{\lambda }\left(y|x\right):=\frac{W{\left(y|x\right)}^{\lambda }Q_{⋆}{\left(y\right)}^{1-\lambda }}{\sum_{y^{\prime }\in Y}W{\left(y^{\prime }|x\right)}^{\lambda }Q_{⋆}{\left(y^{\prime }\right)}^{1-\lambda }},\;\forall \left(x,y\right)\in X\times Y, \end{array}$$

*and where $E_{r}$ was defined in (13).*

In the scenario where users have identical channels toward the base station, one can combine the user identification and decoding stages, i.e., one can distinguish the user’s identity from the decoded message. This appears as the combination R+ν in the bounds in (20).

One could also interpret the result in Theorem 1 as follows. There is one single “big codebook” with MK codewords of length *n*. User 1 uses codewords with indices 1 through *M*; User 2 uses codewords with indices M+1 through 2M, and so forth, concluding with User K using codewords with indices (K−1)M+1 through KM. In each active slot, the receiver decodes the “big codebook”; reliable decoding imposes log(MK)/n=R+ν<I(P,W). However, since in our setting we need to decode K codewords (one per active slot, as opposed to just one), in addition to R+ν≤I(P,W) we also need the bound $\log\left(K\right)/n=\nu <E_{r}\left(\nu +R,P,W\right)$. Comparing the third and fourth bounds of (20) with the per-user probability of error achievability region in [3], we can see an additional ν penalty as a result of a joint probability of error criteria. However, we do not claim (via a matching converse bound) that the rate penalty (at least in the format that we prove) is conclusive.

We should also note that for $\nu <\frac{\alpha }{2}$ (first bound in (20)), with probability approaching one as the blocklength *n* goes to infinity, the users transmit in distinct blocks. Hence, regardless of the achievability scheme, the probability of error under a hypothesis where a collision has occurred is vanishing as the blocklength goes to infinity. As a result, in analyzing the joint probability of error in our achievability scheme, we need to focus on the hypothesis that users do not collide. In our achievability scheme, we use a two-stage decoder which first synchronizes the users (i.e., finds the location of active blocks) and then decodes the users’ messages (which also identifies the users’ identities). The complete proof of Theorem 1 is given in Appendix A.

**Remark** **2.** *The existence of an error exponent bound on the occupancy exponent (i.e., ν) is intuitive (second argument in the second condition in (20)). Even if the decoder has side information about the location of noisy blocks (i.e., blocks with only idle symbols for all users), it still has to decode all the messages within active blocks. Due to independence of the output in different blocks and by the fact that there cannot be intra-block coding, the probability of decoding error over active blocks is asymptotically equal to the sum of the decoding error probabilities in each active block. This puts a restriction on ν based on the best decay rate for the decoding error in each block, i.e., the error exponent.*

### 3.2. Users with Different Choice of Channels

We now move on to a more general setting in which we remove the restriction that all users have identical channels towards the base station (when only one is active). Theorem 3 gives an achievable region when we allow the users’ channels (when they are the sole active user toward the base station) to be chosen from a set of conditional distributions of polynomial size in the blocklength *n*.

In this scenario, one may use the users’ statistics at the channel output to identify the set of users who have a similar channel. In this regard, before introducing Theorem 3, we introduce Theorem 2 in which we characterize the relation between the number of distributions and their pairwise distance for them to be identifiable. The proof of Theorem 2, given in Appendix B, is itself based on a novel graph theoretic technique to analyze the optimal Maximum Likelihood (ML) decoder, which is of independent interest.

**Theorem** **2.** *A sequence of distributions $P=\{P_{1},\ldots ,P_{T_{n}}\}$ is identifiable iff*

(22) $$\begin{array}{c} \lim_{n\to \infty }\sum_{\begin{array}{c} 1\leq i<j\leq T_{n} \end{array}}e^{-2nB(P_{i},P_{j})}=0, \end{array}$$

*where $B(P_{i},P_{j})$ is the Bhatcharrya distance, which was defined in (11).*

**Remark** **3.** *From Theorem 2 one can see that when the number of distributions $T_{n}$ is a constant or grows polynomially with n, the sequence of distributions are always identifiable and by the problem formulation in Section 2.3, it is implied that the probability of error in the identification problem decays to zero as the blocklength n goes to infinity.*

By using the criterion for identifiability of a massive number of distributions in Theorem 2, we move on to the SAS-DM-MAC problem. We adapt the concept of identification of distribution to identify the users’ statistics at the output with an optimal ML test. We use the result of Theorem 2 in the derivation of Theorem 3.

**Theorem** **3.** *For a SAS-DM-MAC where $Q_{i}\left(y|x\right)=W_{c(i)}\left(y|x\right)$ is the channel for user $i\in [K_{n}]$, for $W:=\left\{{⋃}_{i\in [K_{n}]}W_{c_{i}}\right\}\;and\;\left|W\right|=poly\left(n\right)$ the region*

(23) $$\begin{array}{cc} \; & \lim\underset{n\to \infty }{\inf}A_{n}, \end{array}$$

*is achievable where $A_{n}$ is defined as*

(24) $$\begin{array}{cc} A_{n} & :=\underset{\epsilon >0}{⋃}\underset{\begin{array}{c} \left(P_{1},\ldots P_{\left|W\right|}\right)\in P_{X}^{\left|W\right|}\\ \left(\lambda_{1},\ldots \lambda_{\left|W\right|}\right)\in {\left[0,1\right]}^{\left|W\right|} \end{array}}{⋃}\underset{j\in [|W|]}{⋂}\left\{\begin{array}{cc} \nu_{j}+\epsilon & <\frac{\alpha }{2}\\ \nu_{j}+\epsilon & <\min\left\{D\left({\left[P_{j}W_{j}\right]}_{\lambda_{j}}\|\left[P_{j}W_{j}\right]\right),E_{r}\left(R+\nu_{j},P_{j},W_{j}\right)\right\}\\ \alpha +\epsilon & <D({\left[P_{j}W_{j}\right]}_{\lambda_{j}}\|Q_{⋆})\\ 0 & <\underset{\begin{array}{c} i\in [|W|]\\ i\neq j \end{array}}{\inf}B\left(\left[P_{i}W_{i}\right],\left[P_{j}W_{j}\right]\right)\\ R+\nu_{j}+\epsilon & <I(P_{j},W_{j}) \end{array}\right\}, \end{array}$$

*and where $E_{r}$ is defined in (13), $B(P_{i},P_{j})$ was defined in (11) and*

$$\begin{array}{cc} \nu_{j} & :=\frac{1}{n}\log\left(N_{j}\right), \end{array}$$

(25) $$\begin{array}{cc} N_{j} & :=\sum_{i=1}^{K_{n}}1_{\left\{Q_{i}=W_{j}\right\}}\;\;\mathrm{and}\;\mathrm{such}\;\mathrm{that}\;\;\sum_{j=1}^{\left|W\right|}N_{j}=K_{n}, \end{array}$$

(26) $$\begin{array}{cc} {}[P_{j}W_{j}{]}_{\lambda }\left(y\right) & :=\frac{{\left(\left[P_{j}W_{j}\right]\left(y\right)\right)}^{\lambda }{\left(Q_{⋆}\left(y\right)\right)}^{1-\lambda }}{\sum_{y^{\prime }\in Y}{\left(\left[P_{j}W_{j}\right]\left(y^{\prime }\right)\right)}^{\lambda }{\left(Q_{⋆}\left(y^{\prime }\right)\right)}^{1-\lambda }}. \end{array}$$

The proof of Theorem 3 is given in Appendix F.

In the proof of Theorem 3 we propose a three stage decoder which performs the task of synchronization, identification and decoding, sequentially. It is also worth noting that given that the number of users is exponential in the blocklength and the number of channels is only polynomial in the blocklength, there must exist at least a single channel that occupies exponentially many blocks. Hence, it is not surprising to see bounds on the occupancy level of each channel (i.e., $\nu_{j}$) in (24).

**Remark** **4.** *We should note that (as it is apparent in (24)) as long as we have the assumption that the number of user’s channels increase with blocklength n, our optimization space over all users also increases with n and hence our achievability and converse bounds would be dependent on the exact definition of n→∞ (e.g, compared to (20) in Theorem 1). However, in order to compute these bounds, we only need to optimize with respect to $P_{i},i\in \left[K_{n}\right]$ as opposed to $P^{n}$ in the conventional n-letter capacity expressions.*

### 3.3. General Case

Now we investigate a SAS-DM-MAC with no restriction on the channels of the users toward the base station. The key ingredient in our analysis is a novel way to bound the probability of error reminiscent of Gallager’s error exponent [14]. We show an achievability scheme that allows a positive lower bound on the *R* and on ν. This proves that reliable transmission with an exponential number of users with an exponential asynchronous exponent with joint error probability is possible. We use an ML decoder sequentially in each block to identify the active user and its message.

**Theorem** **4.** *For a SAS-DM-MAC the following region is achievable*

(27) $$\begin{array}{c} \lim\underset{n\to \infty }{\inf}\underset{\epsilon >0}{⋃}\underset{\begin{array}{c} \left(P_{1},\ldots P_{K_{n}}\right)\in P_{X}^{K_{n}} \end{array}}{⋃}\underset{i\in [K_{n}]}{⋂}\left\{\begin{array}{cc} \nu & <\frac{\alpha }{2}\\ \nu +R+\epsilon & <C(P_{i},Q_{i},P_{i},Q_{i}),\\ 2\nu +R+\epsilon & <\underset{\begin{array}{c} j\in [K_{n}]\\ j\neq i \end{array}}{\inf}C\left(P_{i},Q_{i},P_{j},Q_{j}\right),\\ \alpha +\nu +R+\epsilon & <C(\;.\;,Q_{⋆},P_{i},Q_{i}), \end{array}\right\} \end{array}$$

The proof of Theorem 4 is given in Appendix G.

**Remark** **5.** *Note that one can show the following (see Appendix H):*

(28a) $$\begin{array}{cc} C(P_{i},Q_{i},P_{i},Q_{i}) & =-\log\sum_{x,x^{\prime },y}P_{i}\left(x\right)P_{i}\left(x^{\prime }\right)\sqrt{Q_{i}\left(y|x\right)Q_{i}\left(y|x^{\prime }\right)}, \end{array}$$

(28b) $$\begin{array}{cc} C(\;.\;,Q_{⋆},P_{i},Q_{i}) & \leq I\left(P_{i},Q_{i}\right)+D\left(\left[P_{i}Q_{i}\right]\|Q_{⋆}\right), \end{array}$$

*where (28a) is the special case of $E_{0}\left(R,P,Q,\rho =1\right)$ in (15), and the right hand side of (28b) is the bound on α+R in the single user strong asynchronous channel [15]. From (28) and (27) we thus see that*

$$\begin{array}{cc} \nu & <E_{r}\left(R,P_{i},Q_{i}\right),\\ \alpha +R & <I\left(P_{i},Q_{i}\right)+D\left(\left[P_{i}Q_{i}\right]\|Q_{⋆}\right)-\nu , \end{array}$$

*which shows that the second and fourth bounds in (27) are, respectively, less than the synchronous channel error exponent and the point-to-point strong asynchronous channel capacity.*

### 3.4. Example

Consider the SAS-DM-MAC with input-output relationship $Y=\sum_{i\in [K_{n}]}X_{i}⊕Z$ with Z∼Bernoulli(δ) for some δ∈(0,1/2). In our notation

$$\begin{array}{cc} Q(y|x)\; & =P\left[X_{i}⊕Z=y|X_{i}=x\right]=P\left[Z=x⊕y\right]\\ & =\left\{\begin{array}{cc} 1-\delta & x⊕y=0\;(i.e.,\;x=y)\\ \delta & x⊕y=1\;(i.e.,\;x\neq y) \end{array}\right.. \end{array}$$

Assume that the input distribution used is P=Bernoulli(p) for some p∈(0,1/2). The achievability region in Theorem 1, is

(29) $$\begin{array}{c} \underset{\begin{array}{c} p\in [0,\frac{1}{2}]\\ \lambda \in [0,1] \end{array}}{⋃}\left\{\begin{array}{cc} \nu & <\alpha /2\\ \nu & <\min\left\{p\cdot d\left(\epsilon_{\lambda }\|\delta \right),\min_{q\in [0,1]}\left\{d\left(\delta \|q\right)+\max\{0,d\left(q\|\frac{1}{2}\right)-R-\nu \right\}\right\}\\ \alpha +R+\nu & <p\cdot d(\epsilon_{\lambda }\|1-\delta )\\ R+\nu & <h(p*\delta )-h(\delta ) \end{array}\right\}, \end{array}$$

where $\epsilon_{\lambda }:=\frac{\delta^{\lambda }{\left(1-\delta \right)}^{\left(1-\lambda \right)}}{\delta^{\lambda }{\left(1-\delta \right)}^{\left(1-\lambda \right)}+{\left(1-\delta \right)}^{\lambda }\delta^{\left(1-\delta \right)}}$.

Moreover, by assuming $P_{i}=\mathrm{Bernoulli}\left(p_{i}\right)$ for all $i\in [K_{n}]$, we can show that the optimal *t* in $C\left(P_{i},Q_{i},P_{j},Q_{j}\right)=\sup_{t}\mu_{i,j}\left(t\right)$ is t=1/2 and hence the achievability region in Theorem 4 is

$$\begin{array}{c} \lim\underset{n\to \infty }{\inf}\underset{\epsilon >0}{⋃}\underset{\begin{array}{c} \left(P_{1},\ldots P_{K_{n}}\right)\in P_{X}^{K_{n}} \end{array}}{⋃}\underset{i\in [K_{n}]}{⋂}\left\{\begin{array}{cc} \nu & <\frac{\alpha }{2}\\ \nu +R+\epsilon & <B\left(P_{i},Q\right)=g\left(p_{i}*p_{i},\delta \right)\\ 2\nu +R+\epsilon & <\underset{i\neq j}{\inf}C\left(P_{i},Q,P_{j},Q\right)=\underset{i\neq j}{\inf}g\left(p_{i}*p_{j},\delta \right)\\ \alpha +\nu +R+\epsilon & <C\left(\;.\;,Q_{⋆},P_{i},Q\right)=g\left(p_{i},\delta \right) \end{array}\right\}, \end{array}$$

where

$$\begin{array}{c} g\left(a,b\right):=-\log\left(1-a+2a\sqrt{b(1-b)}\right). \end{array}$$

Finally, by symmetry, we can see that the optimal $p_{i}=\frac{1}{2},\forall i\in \left[K_{n}\right]$ and hence $g\left(\frac{1}{2},\delta \right)=-\log\left(1/2+\sqrt{\delta (1-\delta )}\right)>0$. So on the BSC(δ) strictly positive rates and ν are achievable. In this regard, the region in Theorem 4 reduces to

(30) $$\begin{array}{c} \alpha +\nu +R<-\log\left(1/2+\sqrt{\delta (1-\delta )}\right). \end{array}$$

**Figure 2:**
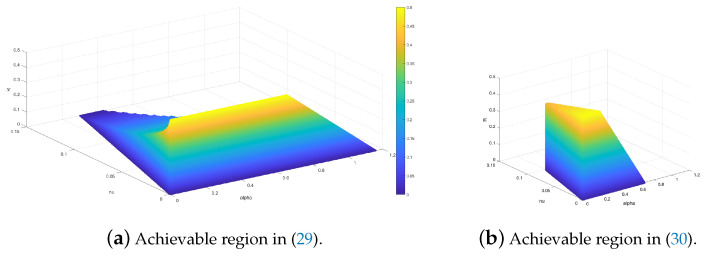
Comparison of the achievable region in Theorems 1 and 4, for the Binary Symmetric Channel with cross over probability δ=0.11.

The achievable region in (29) for (α,ν,R) is shown in Figure 2a. In addition, the achievable region for (α,ν,R) with the achievable scheme in Example (30) is also plotted in Figure 2b for comparison. Figure 2 shows that the achievable scheme in Theorem 1 indeed results in a larger achievable region than the one in Theorem 4.

The fact that the achievability region for Theorem 1 is larger than the achievability region of Theorem 4 for identical channels is not surprising. In Theorem 1 we separated the synchronization and decoding steps, whereas in Theorem 4 synchronization and codeword decoding was done the same time but sequentially for each block. The sequential block decoding step results in a smaller achievability region in Theorem 4.

### 3.5. Converse on the Capacity Region of the SAS-DM-MAC

Thus far, we have provided achievable regions for the SAS-DM-MAC when different users have identical channels; when their channels belong to a set of channels with size that grows polynomially in the blocklength; and without any restriction on the users’ channels. Theorem 5 next provides a converse to the capacity region of the general SAS-DM-MAC.

**Theorem** **5.** *For the SAS-DM-MAC, such that ν<α/2, the following region is impermissible*

(31) $$\begin{array}{c} \lim\underset{n\to \infty }{\inf}\underset{\epsilon >0}{⋃}\underset{\begin{array}{c} \left(P_{1},\ldots P_{K_{n}}\right)\in P_{X}^{K_{n}}\\ \left(\lambda_{1},\ldots \lambda_{K_{n}}\right)\in {\left[0,1\right]}^{K_{n}} \end{array}}{⋂}\left\{\begin{array}{c} \left\{\nu >\frac{1}{K_{n}}\sum_{i=1}^{K_{n}}D({Q_{i}}_{\lambda_{i}}\|Q_{i}\left|P_{i}\right)+\epsilon ,\right.\\ \left.\alpha >\frac{1}{K_{n}}\sum_{i=1}^{K_{n}}D({Q_{i}}_{\lambda_{i}}\|Q_{⋆}\left|P_{i}\right)-\left(1-{\bar{r}}_{n}\right)\left(\nu +R\right)+\epsilon \right\}\\ ⋃\\ \left\{\nu >\frac{1}{K_{n}}\sum_{i=1}^{K_{n}}E_{sp}\left(R,P_{i},Q_{i}\right)+\epsilon \right\}\\ ⋃\\ \left\{R>I(P_{i},Q_{i})+\epsilon \right\} \end{array}\right\}, \end{array}$$

*where ${\bar{r}}_{n}$ is the infimum probability of error, over all estimators T, in distinguishing different hypothesis ${Q_{i}}_{\lambda_{i}}\left(y^{n}|x_{i}^{n}\left(m\right)\right),i\in \left[K_{n}\right],m\in \left[M_{n}\right]$, i.e.,*

(32) $$\begin{array}{c} {\bar{r}}_{n}:=\underset{T}{\inf}\frac{1}{K_{n}M_{n}}\sum_{i=1}^{K_{n}}\sum_{m=1}^{M_{n}}{Q_{i}}_{\lambda_{i}}\left(T\neq i,m|x_{i}^{n}\left(m\right)\right)). \end{array}$$

**Proof.**  The complete proof is given in Appendix I. □

The first bound in (31) corresponds to synchronization, while the second and third bounds correspond to decoding. Also, as it was noted before in Remark 2, one can again see that the value of ν is restricted by the decoding error exponent.

### 3.6. Converse on the Number of Users in a SAS-DM-MAC

In previous sections, we restricted ourselves to the regime where $\nu <\frac{\alpha }{2}$. However, an interesting question is how large a ν can be. Theorem 6 provides a converse bound on the value of ν such that for ν>α, not even reliable synchronization is possible.

**Theorem** **6.** *For a SAS-DM-MAC with ν>α, reliable synchronization is not possible, i.e., even with M=1, one has $P_{e}^{\left(n\right)}>0.$*

The complete proof can be found in Appendix J.

## 4. Discussion and Conclusions

In this paper we studied a Strongly Asynchronous and Slotted Massive Access Channel (SAS-DM-MAC) where $K_{n}:=e^{n\nu }$ different users transmit a randomly selected message among $M_{n}:=e^{nR}$ ones within a strong asynchronous window of length $A_{n}:=e^{n\alpha }$ blocks of *n* channel uses each. We found inner and outer bounds on the (R,α,ν) tuples. Our analysis is based on a joint probability of error in which we required all users’ messages and identities to be jointly correctly decoded at the base station. Our results are focused on the regime $\nu <\frac{\alpha }{2}$, where the probability of user collision vanishes. We proved in Theorem 6 that for the regime ν>α, not even synchronization is possible. We now discuss some of the difficulties in analyzing the region $\frac{\alpha }{2}\leq \nu \leq \alpha$.

For the region $\nu <\frac{\alpha }{2}$, with probability AnKn(An)Kn→1 as blocklength n→∞, the users transmit in distinct blocks. Hence, in analyzing the probability of error of our achievable schemes, we only need to bound the error under the hypothesis that users do not collide. For $\frac{\alpha }{2}\leq \nu \leq \alpha$, we need to consider the events where collisions occur. In particular, based on Lemma 1 (proved in the Appendix L), for $\frac{\alpha }{2}\leq \nu \leq \alpha$, the probability of every arrangement of users is itself vanishing in the blocklength.

**Lemma** **1.** *For the region $\frac{\alpha }{2}\leq \nu \leq \alpha$ the non-colliding arrangement of users has the highest probability among all possible arrangements, yet, the probability of this event is also vanishing as blocklength n goes to infinity.*

As a consequence of Lemma 1, one needs to propose an achievable scheme that accounts for several arrangements (the number of which is non-trivial to the authors) and collision of users and drives the probability of error in these arrangements to zero. It is also worth noting that the number of possible hypotheses is doubly exponential in the blocklength. Finally, it is worth emphasizing that the authors in [16] can get to ν≤α since they require the recovery of the messages of a *large fraction* of users, and require the per-user probability of error to be vanishing (rather than the overall or joint probability of error, which is a much stronger condition). To prove whether or not strictly positive (R,α,ν) are possible in the region $\frac{\alpha }{2}\leq \nu \leq \alpha$, with vanishing joint probability of error, is a challenging open problem.

## Figures and Tables

**Table 1:** Special notation for the identification problem.

$S_{A}$	A symmetric group which is the set of all possible permutations of *A* elements.
$\sigma_{i}$	The *i*-th element of the permutation $\sigma \in S_{A}$; i∈[A].
$K_{k}$	$:=K_{k}\left(w_{\left\{1,2\right\}}\ldots w_{\left\{k,1\right\}}\right)$; A complete graph with *k* nodes and edge weights $w_{\left\{i,j\right\}}$ between node i,j.
$c_{r}^{\left(V\right)},c_{r}^{\left(E\right)}$	A cycle of length *r* represented by the set of its vertices and edges, respectively.
$c_{r}^{\left(V\right)}\left(i\right)$	*i*-th vertex of the cycle $c_{r}^{\left(V\right)}$.
$C_{k}^{\left(r\right)}$	Set of cycles of length *r* in the complete graph $K_{k}$.
$N_{k}^{\left(r\right)}$	Number of cycles of length *r* in a complete graph $K_{k}$.
G(c)	$:=\prod_{\forall i\neq j}w_{i,j}$; Gain of a cycle which we define as the product of the edge weights within the cycle *c*.
AM-GM inequality	Refers to the following inequality $n{\left(\prod_{i=1}^{n}a_{i}\right)}^{\frac{1}{n}}\leq \sum_{i=1}^{n}a_{i}$.

**Figure A1:**
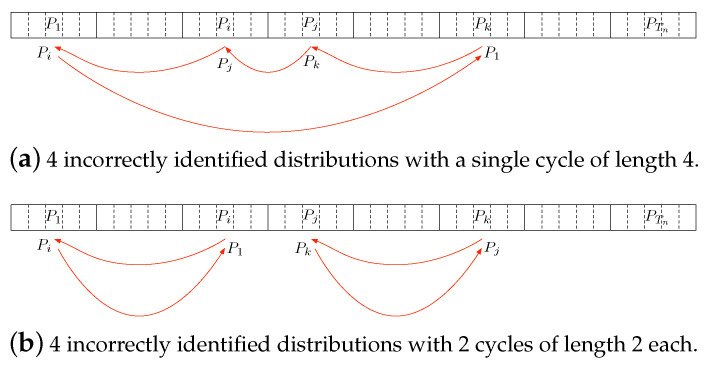
*r* incorrectly identified distribution will form cycles with sum length *r*.

**Figure A2:**
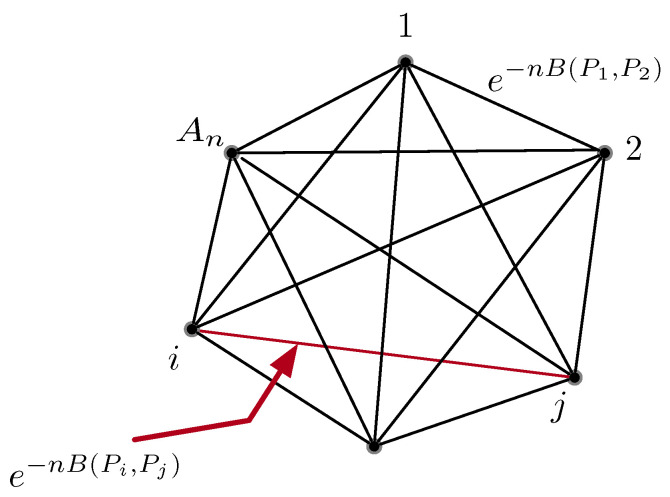
Complete graph $K_{T_{n}}$ with edge weight $e^{-nB(P_{i},P_{j})}$ for every pair of vertices $i\neq j\in [K_{n}]$.

**Figure A3:**
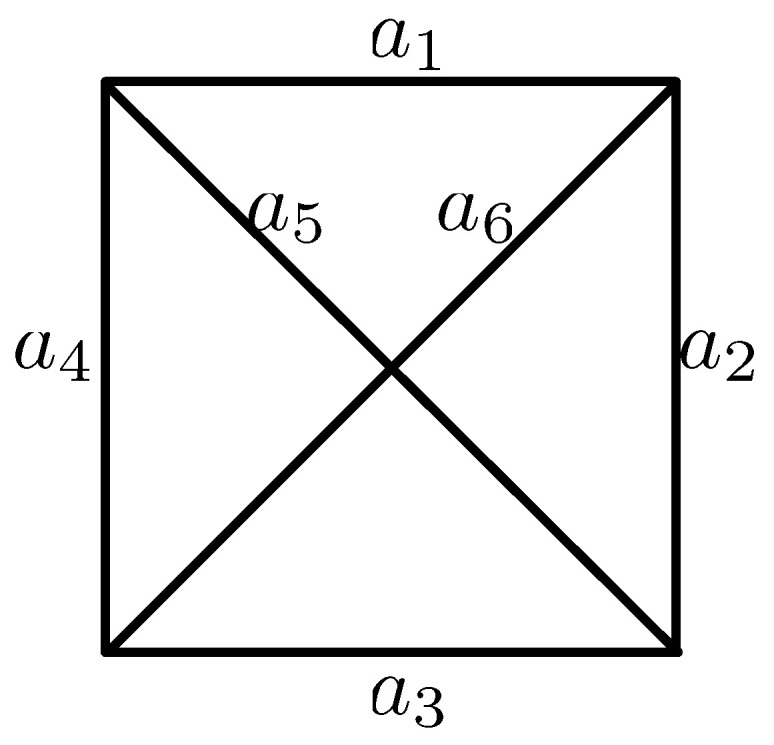
A complete graph with 4 vertices.

**Figure A4:**
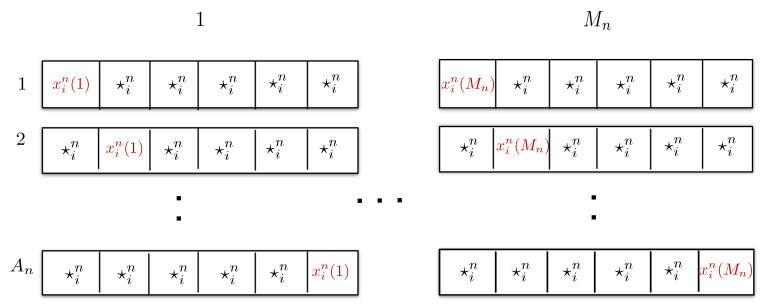
Extended codebook.

## Data Availability

The data presented in this study are available on request from the corresponding author.

## Appendix A. Proof of Theorem 1

We first introduce the codebook generation and then introduce and analyze the decoder.

### Appendix A.1. Codebook Generation

Let $K_{n}=e^{n\nu }$ be the number of users, $A_{n}=e^{n\alpha }$ be the number of blocks, and $M_{n}=e^{nR}$ be the number of messages. Each user $i\in [K_{n}]$ generates a constant composition codebook with composition *P* by drawing each message’s codeword uniformly and independently at random from the *P*-type set T(P) (recall definition in (5)). The codeword of user $i\in [K_{n}]$ for message $m\in [M_{n}]$ is denoted as $x_{i}^{n}\left(m\right)$.

### Appendix A.2. The Decoder and Analysis of the Probability of Error

A two-stage decoder is used, to first synchronize and then decode (which also identifies the users’ identities) the users’ messages. The probability of error of this two-stage decoder is thus given by

PError≤PError|No-collision+Pcollision(A1a)≤PSynchronizationerror|No-collision(A1b)+PDecodingerror|Nosynchronizationerror,No-collision(A1c)+Pcollision.

**Synchronization Step.** We perform a sequential likelihood test as follows. Fix a threshold

(A2) $$\begin{array}{c} T\in [-D(Q_{⋆}\left\|W|P),D(W\right\|Q_{⋆}|P)]. \end{array}$$

For each block $j\in [A_{n}]$ if there exists any message $m\in [M_{n}]$ for any user $i\in [K_{n}]$ such that

(A3) $$\begin{array}{c} L\left(x_{i}^{n}\left(m\right),y_{j}^{n}\right):=\frac{1}{n}\log\frac{W(y_{j}^{n}|x_{i}^{n}\left(m\right))}{Q_{⋆}\left(y_{j}^{n}\right)}\geq T, \end{array}$$

then declare that block *j* is an ‘active’ block, and an ‘idle’ block otherwise. Let

(A4) $$\begin{array}{c} H^{\left(1\right)}:=H_{\left(\left(1,1\right),\left(2,1\right),\ldots ,\left(K_{n},1\right)\right)}, \end{array}$$

be the hypothesis that user *i* (for each $i\in [K_{n}]$) is active in block *i* and sends message $m_{i}=1$. As explained earlier (in the paragraph after Theorem 1), the probability of collision in (A1c) is vanishing and we can focus on calculating (A1a) and (A1b). For (A1a), by symmetry of the different no-collision hypotheses, the average probability of synchronization error (over different such hypotheses) is upper bounded by

(A5) $$\begin{array}{cc} \; & P\left[\mathrm{Synchronization}\;\mathrm{error}|H^{\left(1\right)}\right]\\ \; & \leq \sum_{j=1}^{K_{n}}P\left[\overset{K_{n}}{\underset{i=1}{⋂}}\overset{M_{n}}{\underset{m=1}{⋂}}L\left(x_{i}^{n}\left(m\right),Y_{j}^{n}\right)<T|H^{\left(1\right)}\right]+\sum_{j=K_{n}+1}^{A_{n}}P\left[\overset{K_{n}}{\underset{i=1}{⋃}}\overset{M_{n}}{\underset{m=1}{⋃}}L\left(x_{i}^{n}\left(m\right),Y_{j}^{n}\right)\geq T|H^{\left(1\right)}\right]\\ \; & \leq \sum_{j=1}^{K_{n}}P\left[L\left(x_{j}^{n}\left(1\right),Y_{j}^{n}\right)<T|H^{\left(1\right)}\right]+\sum_{j=K_{n}+1}^{A_{n}}\sum_{i=1}^{K_{n}}\sum_{m=1}^{M_{n}}P\left[L\left(x_{i}^{n}\left(m\right),Y_{j}^{n}\right)\geq T|H^{\left(1\right)}\right]\\ \; & \leq e^{n\nu }e^{-nD\left(W_{\lambda }\left\|W\right|P\right)}+e^{n(\alpha +\nu +R)}e^{-nD\left(W_{\lambda }\left\|Q_{⋆}\right|P\right)}, \end{array}$$

where (A5) can be derived as in Chapter 11 [17]. The upper bound on the probability of error for the synchronization error in (A5) vanishes as *n* goes to infinity if the second and third bound in (20) hold.

**Decoding Step.** In this stage we evaluate the decoding error under the no-collision, no synchronization error hypothesis using a maximum likelihood decoder. Moreover, since in each block, we have to distinguish among $e^{n(R+\nu )}$ messages, we can upper bound the probability of decoding error in (A1b) as

(A6) $$\begin{array}{cc} P[\mathrm{Decoding}\;\mathrm{error}|\;\mathrm{No}\;\mathrm{synchronization}\;\mathrm{error},\;\mathrm{No-collision}] & \leq \sum_{i=1}^{K_{n}}P\left[\mathrm{Decoding}\;\mathrm{error}\;\mathrm{in}\;\mathrm{block}\;i\right]\\ \; & \leq e^{n\nu }e^{-nE_{r}\left(R+\nu ,P,W\right)} \end{array}$$

where (A6) is by [14] and $E_{r}$ is defined in (13). This retrieves the second bound in (20). Finally, as a synchronous channel and since in each block we have to distinguish among $e^{n(R+\nu )}$ messages we retrieve the fourth bound in (20).

## Appendix B. Proof of Theorem 2

We now introduce a graph theoretic Lemma, the proof of which is given in Appendix C.

**Lemma** **A1.**
*In a complete graph $K_{k}\left(w_{1,2},\ldots ,w_{k,1}\right)$, for the set of cycles of length r, $C_{k}^{\left(r\right)}$, we have*

1Nk(r)∑c∈Ck(r)G(c)≤w1,22+…+wk,12k2r2

*where $C_{k}^{\left(r\right)}$ and $N_{k}^{\left(r\right)}$ are the set and number of all cycles of length r in a complete graph $K_{k}$, respectively.*

Next, we use Lemma A1 to derive upper and lower bounds on the probability of error.

### Appendix B.1. Upper Bound on the Probability of Identification Error

We use the optimal ML decoder, which minimizes the average probability of error, given by

(A7) $$\begin{array}{c} \hat{\sigma }\left(x_{1}^{n},\ldots ,x_{T_{n}}^{n}\right):=\arg\max_{\sigma \in S_{T_{n}}}\sum_{i=1}^{T_{n}}\log\left(P_{\sigma_{i}}\left(x_{i}^{n}\right)\right), \end{array}$$

where $P_{\sigma_{i}}\left(x_{i}^{n}\right)=\prod_{t=1}^{n}P_{\sigma_{i}}\left(x_{i,t}\right)$. Let $\hat{\Sigma }=\hat{\sigma }\left(X_{1}^{n},\ldots ,X_{T_{n}}^{n}\right)$ be the estimate of the permutation of the distributions of $X_{i}^{n},i\in \left[T_{n}\right]$. The average probability of error associated with the ML decoder can also be written as

(A8a) $$\begin{array}{cc} P_{e}^{\left(n\right)} & =P\left[\hat{\Sigma }\neq \left[T_{n}\right]\right]\\ \; & =P\left[\underset{\hat{\sigma }\neq \left[T_{n}\right]}{⋃}\hat{\Sigma }=\hat{\sigma }\right]\\ \; & =P\left[\overset{T_{n}}{\underset{r=2}{⋃}}\underset{\begin{array}{c} \hat{\sigma }:\\ \left\{\sum_{i=1}^{T_{n}}1_{\left\{{\hat{\sigma }}_{i}\neq i\right\}}=r\right\} \end{array}}{⋃}\hat{\Sigma }=\hat{\sigma }\right] \end{array}$$

(A8b) $$\begin{array}{cc} \; & =P\left[\overset{T_{n}}{\underset{r=2}{⋃}}\underset{\begin{array}{c} \hat{\sigma }:\\ \left\{\sum_{i=1}^{T_{n}}1_{\left\{{\hat{\sigma }}_{i}\neq i\right\}}=r\right\} \end{array}}{⋃}\sum_{i=1}^{T_{n}}\log\frac{P_{{\hat{\sigma }}_{i}}\left(X_{i}^{n}\right)}{P_{i}\left(X_{i}^{n}\right)}\geq 0\right], \end{array}$$

where P over $\left(X_{1}^{n},...,X_{T_{n}}^{n}\right)$ is used when $X_{i}^{n}\overset{i.i.d}{∼}P_{\sigma_{i}},\forall i\in \left[T_{n}\right]$ and where (A8a) is due to the requirement that each sequence is distributed according to a distinct distribution and hence the number of incorrect distributions ranges from $\left[2:T_{n}\right]$. In order to avoid considering the same set of error events multiple times in the union over $\left\{\hat{\sigma }:\sum_{i=1}^{T_{n}}1_{\left\{{\hat{\sigma }}_{i}\neq i\right\}}=r\right\}$, we incorporate a graph theoretic interpretation of $\left\{\sum_{i=1}^{T_{n}}1_{\left\{{\hat{\Sigma }}_{i}\neq i\right\}}=r\right\}$ in (A8b) which is used to denote the fact that we have identified *r* distributions incorrectly. It is apparent from Figure A1a that the event that *r* distributions are incorrectly identified either forms a single cycle of length *r* (r=4 in Figure A1a) or multiple cycles with sum length equal to *r* (2 cycles of length 2 in Figure A1b). Hence, it is straightforward to show that we can replace the union over $\left\{\hat{\sigma }:\sum_{i=1}^{T_{n}}1_{\left\{{\hat{\sigma }}_{i}\neq i\right\}}=r\right\}$ in (A8b) with the union over cycles of length *r* in a complete graph $K_{T_{n}}$, i.e., $\left\{c\in C_{T_{n}}^{\left(r\right)}\right\}$.

Hence, we can re-write the probability of error in (A8b) as

(A9) $$\begin{array}{cc} P_{e}^{\left(n\right)} & =P\left[\overset{T_{n}}{\underset{r=2}{⋃}}\underset{\begin{array}{c} c\in C_{T_{n}}^{\left(r\right)} \end{array}}{⋃}\sum_{i\in c^{\left(V\right)}}\log\frac{P_{c^{\left(V\right)}\left(i\right)}\left(X_{i}^{n}\right)}{P_{i}\left(X_{i}^{n}\right)}\geq 0\right]\\ \; & \leq \sum_{r=2}^{T_{n}}\sum_{\begin{array}{c} c\in C_{T_{n}}^{\left(r\right)} \end{array}}e^{-n\sum_{i\in c^{\left(V\right)}}B\left(P_{c^{\left(V\right)}\left(i\right)},P_{i}\right)}, \end{array}$$

where the inequality in (A9) follows from Appendix D. Next, we define $w_{i,j}:=e^{-nB(P_{i},P_{j})}$ to be the edge weight between vertices (i,j) in the complete graph $K_{T_{n}}\left(w_{\left(1,2\right)},\ldots w_{\left(K_{n},1\right)}\right)$ shown in Figure A2.

With this definition of the complete graph $K_{T_{n}}$, it is easy to see that $G\left(c\right)=e^{-n\sum_{i\in c^{\left(V\right)}}B\left(P_{c^{\left(V\right)}\left(i\right)},P_{i}\right)}$ is the gain of cycle *c* in the complete graph $K_{T_{n}}$. Based on this, we re-write the probability of error in (A9) as

(A10a)Pe(n)≤∑r=2Tn∑c∈CAn(r)G(c)(A10b)≤∑r=2TnNTn(r)Tn2r2w1,22+…+wTn,12r/2(A10c)≤∑r=2Tn4r∑1≤i<j≤Tne−2nB(Pi,Pj)r/2(A10d)≤16∑1≤i<j≤Tne−2nB(Pi,Pj)1−4∑1≤i<j≤Tne−2nB(Pi,Pj),

where *r* enumerates the number of incorrectly identified distributions and where (A10b) is by Lemma A1 and (A10c) is by Fact 1 (see Appendix C) and

NTn(r)nTnr/2=Tnr(r−1)!/2Tn2r/2≤4r.

Finally, (A10d) will go to zero as *n* goes to infinity if

$$\lim_{n\to \infty }\sum_{\begin{array}{c} 1\leq i<j\leq T_{n} \end{array}}e^{-2nB(P_{i},P_{j})}=0.$$

**Remark** **A1.**
*As a result of Lemma A1, it can be seen from (A10c) that the sum of probabilities that r≥3 distributions are incorrectly identified is dominated by the probability that only r=2 distributions are incorrectly identified. This shows that the most probable error event is indeed an error event with two wrong distributions.*

### Appendix B.2. Lower Bound on the Probability of Identifiability Error

For our converse, we use the optimal ML decoder, and as a lower bound to the probability of error in (A8b), we only consider the set of error events with exactly two incorrect distributions, i.e., the set of events with r=2. In this case we have

(A11) $$\begin{array}{cc} P_{e}^{\left(n\right)} & \geq P\left[\underset{\begin{array}{c} 1\leq i<j\leq T_{n} \end{array}}{⋃}\log\frac{P_{i}\left(X_{j}^{n}\right)}{P_{j}\left(X_{j}^{n}\right)}+\log\frac{P_{j}\left(X_{i}^{n}\right)}{P_{i}\left(X_{i}^{n}\right)}\geq 0\right]\\ & \geq \frac{{\left(\sum_{\begin{array}{c} 1\leq i<j\leq T_{n} \end{array}}P\left[\xi_{i,j}\right]\right)}^{2}}{\sum_{\begin{array}{c} \left(i,j),(j,k\right)\\ \left(i,j)\neq (l,k\right)\\ i\neq j,l\neq k \end{array}}P\left[\xi_{i,j},\xi_{k,l}\right]}, \end{array}$$

where P is taken over $\left(X_{1}^{n},...,X_{T_{n}}^{n}\right)$ and is used when $X_{i}^{n}\overset{i.i.d}{∼}P_{\sigma_{i}},\forall i\in \left[T_{n}\right]$ and where (A11) is by [18] and finally

(A12) $$\begin{array}{cc} \; & \xi_{i,j}:=\left\{\log\frac{P_{i}}{P_{j}}\left(X_{j}^{n}\right)+\log\frac{P_{j}}{P_{i}}\left(X_{i}^{n}\right)\geq 0\right\}. \end{array}$$

We prove in Appendix E that a lower bound on $P_{e}^{\left(n\right)}$ is given by

(A13a)Pe(n)≥∑1≤i<j≤Tne−2nB(Pi,Pj)2∑1≤i<j<k≤Tne−nB(Pi,Pj)−nB(Pi,Pk)−nB(Pk,Pj)+∑1≤i<j≤Tne−2nB(Pi,Pj)2(A13b)≥∑1≤i<j≤Tne−2nB(Pi,Pj)28∑1≤i<j≤Tne−2nB(Pi,Pj)32+∑1≤i<j≤Tne−2nB(Pi,Pj)2(A13c)=∑1≤i<j≤Tne−2nB(Pi,Pj)8+∑1≤i<j≤Tne−2nB(Pi,Pj),

where (A13b) is by Lemma A1. As can be seen from (A13c), if $\lim_{n\to \infty }\sum_{\begin{array}{c} 1\leq i<j\leq T_{n} \end{array}}e^{-2nB(P_{i},P_{j})}\neq 0$, the probability of error is bounded away from zero. As a result

$$\lim_{n\to \infty }\sum_{\begin{array}{c} 1\leq i<j\leq T_{n} \end{array}}e^{-2nB(P_{i},P_{j})}=0,$$

must hold, which also matches our upper bound on the probability of error in (A10d).

## Appendix C. Proof of Lemma A1

We first consider the case that *r* is an even number and then prove

(A14) $$\begin{array}{c} r{\left(n\right)}^{\frac{r}{2}-1}\left(G\left(c_{1}\right)+\ldots G\left(c_{N}\right)\right)\leq \frac{Nr}{n}{\left(a_{1}^{2}+\ldots +{a_{n}}^{2}\right)}^{\frac{r}{2}}, \end{array}$$

where as shorthand notation we defined and use $N:=N_{k}^{\left(r\right)}$ and n:=k2 and $\left\{w_{1,2}\ldots w_{k,1}\right\}=\left\{a_{1},\ldots ,a_{n}\right\}$.

Our goal is to expand the right hand side (RHS) of (A14) such that all elements have coefficient 1. Then, we parse these elements into *N* groups (details provided later) such that using the AM-GM inequality (defined in Table 1) on each group, we obtain one of the *N* terms on the LHS of (A14). Before stating the rigorous proof and to shed some light onto the proof strategy, we provide an example for the graph with k=4 vertices shown in Figure A3. In this example, we consider the Lemma for r=4 cycles (for which N=3).

We may expand the RHS in (A14) as

$$\begin{array}{cc} \; & \;2{\left(a_{1}^{2}+\ldots +a_{6}^{2}\right)}^{2}=\Theta_{1}+\Theta_{2}+\Theta_{3},\\ \; & \Theta_{1}\;=\;\{a_{1}^{4}+a_{2}^{4}+a_{3}^{4}+a_{4}^{4}+a_{1}^{2}a_{3}^{2}+a_{1}^{2}a_{3}^{2}+a_{2}^{2}a_{4}^{2}+a_{2}^{2}a_{4}^{2}\\ \; & +a_{1}^{2}a_{2}^{2}+a_{1}^{2}a_{2}^{2}+a_{1}^{2}a_{2}^{2}+a_{1}^{2}a_{2}^{2}+a_{1}^{2}a_{4}^{2}+a_{1}^{2}a_{4}^{2}+a_{1}^{2}a_{4}^{2}+a_{1}^{2}a_{4}^{2}\\ & +a_{2}^{2}a_{3}^{2}+a_{2}^{2}a_{3}^{2}+a_{2}^{2}a_{3}^{2}+a_{2}^{2}a_{3}^{2}+a_{3}^{2}a_{4}^{2}+a_{3}^{2}a_{4}^{2}+a_{3}^{2}a_{4}^{2}+a_{3}^{2}a_{4}^{2}\}\\ & \Theta_{2}\;=\;\{a_{1}^{4}+a_{6}^{4}+a_{3}^{4}+a_{5}^{4}+a_{5}^{2}a_{6}^{2}+a_{5}^{2}a_{6}^{2}+a_{1}^{2}a_{3}^{2}+a_{1}^{2}a_{3}^{2}\\ & +a_{1}^{2}a_{6}^{2}+a_{1}^{2}a_{6}^{2}+a_{1}^{2}a_{6}^{2}+a_{1}^{2}a_{6}^{2}+a_{1}^{2}a_{5}^{2}+a_{1}^{2}a_{5}^{2}+a_{1}^{2}a_{5}^{2}+a_{1}^{2}a_{5}^{2}\\ & +a_{3}^{2}a_{6}^{2}+a_{3}^{2}a_{6}^{2}+a_{3}^{2}a_{6}^{2}+a_{3}^{2}a_{6}^{2}+a_{3}^{2}a_{5}^{2}+a_{3}^{2}a_{5}^{2}+a_{3}^{2}a_{5}^{2}+a_{3}^{2}a_{5}^{2}\}\\ & \Theta_{3}\;=\;\{a_{4}^{4}+a_{5}^{4}+a_{2}^{4}+a_{6}^{4}+a_{5}^{2}a_{6}^{2}+a_{5}^{2}a_{6}^{2}+a_{2}^{2}a_{4}^{2}+a_{2}^{2}a_{4}^{2}\\ & +a_{4}^{2}a_{5}^{2}+a_{4}^{2}a_{5}^{2}+a_{4}^{2}a_{5}^{2}+a_{4}^{2}a_{5}^{2}+a_{4}^{2}a_{6}^{2}+a_{4}^{2}a_{6}^{2}+a_{4}^{2}a_{6}^{2}+a_{4}^{2}a_{6}^{2}\\ & +a_{2}^{2}a_{5}^{2}+a_{2}^{2}a_{5}^{2}+a_{2}^{2}a_{5}^{2}+a_{2}^{2}a_{5}^{2}+a_{2}^{2}a_{6}^{2}+a_{2}^{2}a_{6}^{2}+a_{2}^{2}a_{6}^{2}+a_{2}^{2}a_{6}^{2}\}. \end{array}$$

If we use the AM-GM inequality on $\Theta_{1}$, $\Theta_{2}$ and $\Theta_{3}$, we can obtain the lower bound equal to $24\left(a_{1}a_{2}a_{3}a_{4}\right),24\left(a_{1}a_{6}a_{3}a_{5}\right)$ and $24(a_{4}a_{5}a_{2}a_{6})$, respectively where $rn^{\frac{r}{2}-1}=24$ and hence (A14) holds in this example.

We proceed to prove Lemma A1 for arbitrary *k* and (even) r≥2. We propose the following scheme to group the elements on the RHS of (A14) and then prove that this grouping indeed leads to the claimed inequality.

### Grouping Scheme

For each cycle $c_{i}=\left\{a_{i_{1}}\ldots ,a_{i_{r}}\right\}$, we need a group of elements, $\Theta_{i}$, from the RHS of (A14). In this regard, we consider all possible subsets of the edges of cycle $c_{i}$ with 1 to $\frac{r}{2}$ elements (e.g., $\left\{\left\{a_{i_{1}}\right\},\ldots \left\{a_{i_{1}},a_{i_{2}}\right\},\ldots \left\{a_{i_{1}}\ldots ,a_{i_{r/2}}\right\},\ldots \right\}$). For each one of these subsets, we find the respective elements from the RHS of (A14) that is the multiplication of the elements in that subset. For example, for the subset $\left\{a_{i_{1}},a_{i_{2}},a_{i_{3}}\right\}$, we consider the elements like $a_{i_{1}}^{n_{i_{1}}}a_{i_{2}}^{n_{i_{2}}}a_{i_{3}}^{n_{i_{3}}}$ for all possible $n_{i_{1}},n_{i_{2}},n_{i_{3}}>0$ from the RHS of (A14). However, note that we do not assign all such elements to cycle $c_{i}$ only. If there are *ℓ* cycles of length *r* that all contain $\left\{a_{i_{1}},a_{i_{2}},a_{i_{3}}\right\}$, we should assign $\frac{1}{\ell }$ of the elements like $a_{i_{1}}^{n_{i_{1}}}a_{i_{2}}^{n_{i_{2}}}a_{i_{3}}^{n_{i_{3}}},\;n_{i_{1}},n_{i_{2}},n_{i_{3}}>0$ to cycle $c_{i}$ (note the symmetry of the strategy over cycles).

We state some facts, which can be easily.

**Fact 1.** In a complete graph $K_{k}$, there are N=Nk(r)=kr(r−1)!2 cycles of length *r*.

**Fact 2.** By expanding the RHS of (A14) such that all elements have coefficient 1, we end up with $\left(\frac{N_{k}^{\left(r\right)}r}{n}\right)n^{\frac{r}{2}}$ elements.

**Fact 3.** Expanding the RHS of (A14) such that all elements have coefficient 1, and finding their product yields

$${\left(a_{1}\times \ldots \times a_{n}\right)}^{\left(\frac{Nr}{n}\right)rn^{\frac{r}{2}-1}}.$$

**Fact 4.** In the above grouping scheme each element on the RHS of (A14) is summed in exactly one group. Hence, by symmetry and Fact 2, each group is the sum of $rn^{\frac{r}{2}-1}$ elements.

Now, consider any two cycles $c_{i}=\left\{a_{i_{1}},\ldots ,a_{i_{r}}\right\},c_{j}=\left\{a_{j_{1}},\ldots ,a_{j_{r}}\right\}$. Assume that using the above grouping scheme, we obtain the group of elements $\Theta_{i},\Theta_{j}$ (where by fact 3 each one is the sum of $rn^{\frac{r}{2}-1}$ elements). If we apply the AM-GM inequality on each of the two groups, we obtain

$$\begin{array}{c} \Theta_{i}\geq rn^{\frac{r}{2}-1}{\left(a_{i_{1}}^{n_{i_{1}}}\times \ldots \times a_{i_{r}}^{n_{1_{r}}}\right)}^{\left(\frac{1}{rn^{\frac{r}{2}-1}}\right)},\\ \Theta_{j}\geq rn^{\frac{r}{2}-1}{\left(a_{j_{1}}^{n_{j_{1}}}\times \ldots \times a_{j_{r}}^{n_{j_{r}}}\right)}^{\left(\frac{1}{rn^{\frac{r}{2}-1}}\right)}, \end{array}$$

where $\prod_{t=1}^{r}a_{i_{t}}^{n_{i_{t}}}$ is the product of the elements in $\Theta_{i}$. By symmetry of the grouping scheme for different cycles, it is obvious that $\forall t\in \left[r\right],n_{i_{t}}=n_{j_{t}}$. Hence $n_{i_{t}}=n_{j_{t}}=p_{t},\forall i,j\in \left[N\right]$. i.e., we have

(A15) $$\begin{array}{cc} \Theta_{i} & \geq rn^{\frac{r}{2}-1}{\left(a_{i_{1}}^{p_{1}}\times \ldots \times a_{i_{r}}^{p_{r}}\right)}^{\left(\frac{1}{rn^{\frac{r}{2}-1}}\right)}. \end{array}$$

By symmetry of the grouping scheme over the elements of each cycle, we also get that $n_{i_{k}}=n_{i_{l}}=q_{i},\forall k,l\in \left[r\right]$, i.e.,

(A16) $$\begin{array}{c} \Theta_{i}\geq rn^{\frac{r}{2}-1}{\left(a_{i_{1}}^{q_{i}}\times \ldots \times a_{i_{r}}^{q_{i}}\right)}^{\left(\frac{1}{rn^{\frac{r}{2}-1}}\right)}. \end{array}$$

It can be seen from (A15) and (A16) that all elements of all groups have the same power $n_{i_{t}}=p,\forall i\in \left[N\right],t\in \left[r\right]$. i.e.,

$$\begin{array}{cc} \Theta_{i} & \geq rn^{\frac{r}{2}-1}{\left(a_{i_{1}}^{p}\times \ldots \times a_{i_{r}}^{p}\right)}^{\left(\frac{1}{rn^{\frac{r}{2}-1}}\right)}. \end{array}$$

Since each element on the RHS of (A14) is assigned to one and only one group and since $\prod_{t=1}^{r}a_{i_{t}}^{n_{i_{t}}}=\prod_{t=1}^{r}a_{i_{t}}^{p}$ is the product of the elements of each group $\Theta_{i}$, the product of all elements in $\Theta_{1}+\ldots +\Theta_{N}$ (which is equal to product of the elements in the expanded version of the RHS of (A14)) is $\prod_{i=1}^{N}\prod_{t=1}^{r}a_{i_{t}}^{p}$.

In addition, since each $a_{i}$ appears in exactly $\frac{Nr}{n}$ of the cycles, by Fact 3 and a double counting argument, we have

$$p\times \frac{Nr}{n}=\left(\frac{Nr}{n}\right)rn^{\frac{r}{2}-1},$$

and hence $p=rn^{\frac{r}{2}-1}$. Hence, the lower bound of the AM-GM inequality on the $\Theta_{1}+\ldots +\Theta_{N}$, will result in

$$rn^{\frac{r}{2}-1}G\left(c_{1}\right)+\ldots +rn^{\frac{r}{2}-1}G\left(c_{N_{k}^{\left(r\right)}}\right),$$

and the Lemma is proved for even *r*.

For odd values of *r*, the problem that may arise by using the grouping strategy in its current form, is when $r<\frac{k}{2}$. In this case, some of the terms on the RHS of (A14) may contain multiplication of $a_{i}$’s that are not present in any of the $G(c_{i})$’s. To overcome this, take both sides to the power of 2m for the smallest *m* such that $rm>\frac{k}{2}$. Then the RHS of (A14) is at most the multiplication of rm different $a_{i}$’s and on the LHS of (A14), there are 2m cycles of length *r* multiplied together. By our choice of 2m, now, all possible combinations of $a_{i}$’s on the RHS are present in at least one cycle multiplication in the LHS. Hence, it is straightforward to use the same strategy as even values of *r* to prove the theorem for the odd values of *r*.

## Appendix D. Proof of (A9)

The inequality in (A9) is by

(A17a)P∑i∈c(V)logPc(V)(i)XinPiXin≥0≤expninftlogE∏i∈c(V)Pc(V)(i)XinPiXint(A17b)≤expn∑i∈c(V)logEPc(V)(i)XinPiXin1/2=exp−n∑i∈c(V)B(Pc(V)(i),Pi),

where (A17a) is by the Chernoff inequality. The fact that we used t=1/2 in (A17b) instead of finding the exact optimizing *t*, comes from the fact that t=1/2 is the optimal choice for r=2 and as we will explain in Remark A1. The rest of the error events are dominated by the set of only 2 incorrectly identified distributions. This can be seen as follows for $X_{1}^{n}\overset{i.i.d}{∼}P_{1},X_{2}^{n}\overset{i.i.d}{∼}P_{2}$

PlogP1(X2n)P2(X2n)+logP2(X1n)P1(X1n)≥0=∑P^1,P2^:∑x∈XP^1(x)logP2(x)P1(x)+P2^(x)logP1(x)P2(x)≥0exp−nDP^1‖P1−nDP^2‖P2(A18)≐e−nDP˜‖P1−nDP˜‖P2=e−2nB(P1,P2),

where $\tilde{P}$ in the first equality in (A18), by using the Lagrangian method, can be shown to be equal to $\tilde{P}\left(x\right)=\frac{\sqrt{P_{1}\left(x\right)P_{2}\left(x\right)}}{\sum_{x^{\prime }}\sqrt{P_{1}\left(x^{\prime }\right)P_{2}\left(x^{\prime }\right)}}$ and subsequently the second inequality in (A18) is proved.

## Appendix E. Proof of (A13a)

We upper bound the denominator of (A11) by

$$\begin{array}{cc} \; & \;P\left[\xi_{i,j},\xi_{i,k}\right]=P\left[\log\frac{P_{i}\left(X_{j}^{n}\right)}{P_{j}\left(X_{j}^{n}\right)}+\log\frac{P_{j}\left(X_{i}^{n}\right)}{P_{i}\left(X_{i}^{n}\right)}\geq 0\cap \;\log\frac{P_{i}\left(X_{k}^{n}\right)}{P_{k}\left(X_{k}^{n}\right)}+\log\frac{P_{k}\left(X_{i}^{n}\right)}{P_{i}\left(X_{i}^{n}\right)}\geq 0\right]\\ & \leq P\left[\log\frac{P_{i}\left(X_{j}^{n}\right)}{P_{j}\left(X_{j}^{n}\right)}+\log\frac{P_{j}\left(X_{i}^{n}\right)}{P_{i}\left(X_{i}^{n}\right)}+\log\frac{P_{i}\left(X_{k}^{n}\right)}{P_{k}\left(X_{k}^{n}\right)}+\log\frac{P_{k}\left(X_{i}^{n}\right)}{P_{i}\left(X_{i}^{n}\right)}\geq 0\right]\\ & \leq \exp\left\{n\underset{t}{\inf}\log\left(E\left[{\left(\;\frac{P_{i}\left(X_{j}^{n}\right)}{P_{j}\left(X_{j}^{n}\right)}\cdot \frac{P_{j}\left(X_{i}^{n}\right)}{P_{i}\left(X_{i}^{n}\right)}\cdot \frac{P_{i}\left(X_{k}^{n}\right)}{P_{k}\left(X_{k}^{n}\right)}\cdot \frac{P_{k}\left(X_{i}^{n}\right)}{P_{i}\left(X_{i}^{n}\right)}\right)}^{t}\right]\right)\right\}\\ & \;\;\leq \;\exp\left\{\;n\log E\left[\;{\left(\frac{P_{i}\left(X_{j}^{n}\right)}{P_{j}\left(X_{j}^{n}\right)}\;\cdot \;\frac{P_{j}\left(X_{i}^{n}\right)}{P_{i}\left(X_{i}^{n}\right)}\cdot \;\frac{P_{i}\left(X_{k}^{n}\right)}{P_{k}\left(X_{k}^{n}\right)}\cdot \;\frac{P_{k}\left(X_{i}^{n}\right)}{P_{i}\left(X_{i}^{n}\right)}\;\right)}^{\frac{1}{2}}\;\right]\right\} \end{array}$$

(A19) $$\begin{array}{c} =\exp\left\{-nB\left(P_{i},P_{j}\right)-nB\left(P_{j},P_{k}\right)-nB\left(P_{i},P_{k}\right)\right\}. \end{array}$$

An upper bound for $P\left[\xi_{i,j},\xi_{k,l}\right]$ can be derived similarly.

## Appendix F. Proof of Theorem 3

We propose a three-stage achievability scheme where we should again note that with similar arguments as the ones in Theorem 1, by imposing the first bound in (24), we can mainly focus on the no-collision hypothesis. The three achievability stages perform the tasks of synchronization, identification, and decoding, respectively and the joint probability of error can be decomposed as

$$\begin{array}{cc} P\left[\mathrm{Error}\right]\; & \leq P\left[\;\mathrm{Synchronization}\;\mathrm{error}\;|\;\mathrm{No-collision}\;\right]\\ & +P\left[\;\mathrm{Identification}\;\mathrm{error}\;|\;\mathrm{No}\;\mathrm{synchronization}\;\mathrm{error},\;\mathrm{No-collision}\;\right]\\ & +P\left[\;\mathrm{Decoding}\;\mathrm{error}\;|\;\mathrm{No}\;\mathrm{synchronization},\;\mathrm{No}\;\mathrm{identification}\;\mathrm{error},\;\mathrm{No-collision}\right]\\ \; & +P\left[\mathrm{collision}\right]. \end{array}$$

### Appendix F.1. Codebook Generation

Let $K_{n}=e^{n\nu }$ be the number of users, $A_{n}=e^{n\alpha }$ be the number of blocks, $M_{n}=e^{nR}$ be the number of messages, and |W|=poly(n) be the number of channels. Each user $i\in [K_{n}]$ generates a random i.i.d codebook according to distribution $P_{c(i)}$ where the index c(i)∈[|W|] is chosen based on the channel $Q_{i}=W_{c(i)}$. For each user $i\in [K_{n}]$, the codeword for each message $m\in [M_{n}]$ is denoted as $x_{i}^{n}\left(m\right)$.

### Appendix F.2. Probability of Error Analysis

A three-stage decoder is used. We now introduce the three stages and bound the probability of error for each stage.

**Synchronization Step.** We perform a sequential likelihood ratio test for synchronization as follows. Recall $Q_{i}\left(\cdot |\cdot \right)=W_{c(i)}\left(\cdot |\cdot \right)$ for all user $i\in [K_{n}]$. Fix thresholds

(A20) $$\begin{array}{c} T_{c(i)}\in \left[-D\left(Q_{⋆}\|\left[P_{c(i)}W_{c(i)}\right]\right),D\left(\left[P_{c(i)}W_{c(i)}\right]\|Q_{⋆}\right)\right],\;i\in \left[K_{n}\right]. \end{array}$$

For each block $j\in [A_{n}]$ if there exists any user $i\in [K_{n}]$ such that

(A21) $$\begin{array}{c} L_{c(i)}\left(y_{j}^{n}\right):=\frac{1}{n}\log\frac{\left[P_{c(i)}W_{c(i)}\right]\left(y_{j}^{n}\right)}{Q_{⋆}\left(y_{j}^{n}\right)}\geq T_{c(i)}, \end{array}$$

then declare that block *j* is an ‘active’ block. Else, declare that block *j* is an ‘idle’ block. Note that were able to calculate the probabilities of error corresponding to (A3) by leveraging the constant composition construction of codewords in Theorem 1. In here, we can leverage the i.i.d. construction of the codewords and calculate the probability of error corresponding to (A21).

We now find an upper bound on the average probability of synchronization error for this scheme over different hypotheses. With the same argument as the one after (A4) we have

$$\begin{array}{cc} \; & P\left[\mathrm{Synchronization}\;\mathrm{error}|H^{\left(1\right)}\right]\\ \; & \leq P\left[\overset{K_{n}}{\underset{i=1}{⋃}}L_{c(i)}\left(Y_{i}^{n}\right)<T_{c(i)}|H^{\left(1\right)}\right]+P\left[\overset{A_{n}}{\underset{s=K_{n}+1}{⋃}}\overset{K_{n}}{\underset{i=1}{⋃}}L_{c(i)}\left(Y_{s}^{n}\right)\geq T_{c(i)}|H^{\left(1\right)}\right]\\ \; & \leq \sum_{i=1}^{K_{n}}P\left[L_{c(i)}\left(Y_{i}^{n}\right)<T_{c(i)}|H^{\left(1\right)}\right]+e^{n\alpha }P\left[\overset{K_{n}}{\underset{i=1}{⋃}}L_{c(i)}\left(Y^{n}\right)\geq T_{c(i)}|H^{\left(1\right)}\right] \end{array}$$

(A22a) $$\begin{array}{cc} \; & =\sum_{i=1}^{K_{n}}P\left[L_{c(i)}\left(Y_{i}^{n}\right)<T_{c(i)}|H^{\left(1\right)}\right]+e^{n\alpha }P\left[\overset{\left|W\right|}{\underset{j=1}{⋃}}L_{j}\left(Y^{n}\right)\geq T_{j}|H^{\left(1\right)}\right] \end{array}$$

(A22b) $$\begin{array}{cc} \; & \leq \sum_{i=1}^{K_{n}}e^{-nD\left({\left[P_{c(i)}W_{c(i)}\right]}_{\lambda_{i}}\|\left[P_{c(i)}W_{c(i)}\right]\right)}+e^{n\alpha }\sum_{j=1}^{\left|W\right|}e^{-nD\left({\left[P_{j}W_{j}\right]}_{\lambda_{j}}\|Q_{⋆}\right)} \end{array}$$

(A22c) $$\begin{array}{cc} \; & =\sum_{j=1}^{\left|W\right|}N_{j}e^{-nD\left({\left[P_{j}W_{j}\right]}_{\lambda_{j}}\|\left[P_{j}W_{j}\right]\right)}+e^{n\alpha }e^{-nD\left({\left[P_{j}W_{j}\right]}_{\lambda_{j}}\|Q_{⋆}\right)}, \end{array}$$

where (A22a) is by taking j:=c(i) and (A22b) is by the Chernoff inequality in which ${\left[P_{j}W_{j}\right]}_{\lambda }$ was defined in (26) and where (A22c) is again by taking j:=c(i) and re-counting the events.

The probability of error in this stage will decay to zero as blocklength *n* goes to infinity if for all j∈[|W|] and for a ϵ>0

(A23a) $$\begin{array}{cc} \nu_{j}+\epsilon & <D\left({\left[P_{j}W_{j}\right]}_{\lambda_{j}}\|\left[P_{j}W_{j}\right]\right), \end{array}$$

(A23b) $$\begin{array}{cc} \alpha +\epsilon & <D\left({\left[P_{j}W_{j}\right]}_{\lambda_{j}}\|Q_{⋆}\right). \end{array}$$

This corresponds to the second and third bounds in (24).

**Identification Step.** Having found the location of the ‘active’ blocks, we move on to the second stage of the achievability scheme to identify which user is active in which block. We note that, by the random codebook generation and the memoryless property of the channel, the output of the block occupied by user $i\in [K_{n}]$ is i.i.d distributed according to the marginal distribution

$$\begin{array}{c} \left[P_{c(i)}Q_{c(i)}\right]\left(y\right):=\sum_{x\in X}P_{c(i)}\left(x\right)Q_{c(i)}\left(y|x\right). \end{array}$$

We leverage this property and customize the result in Theorem 2 to identify the different distributions of the different users. Note that at this point, we only distinguish the users with different channels from one another. Users with the same channel are distinguished from each other in the decoding stage as will be explained next. In Theorem 2, it was assumed that all the distributions are distinct, while in here, our distributions are not necessarily distinct. The only modification that is needed in order to use the result of Theorem 2 is as follows. We need to consider a graph in which the edge between every two similar distributions has edge weights equal to zero (as opposed to $e^{B(P,P)}=e^{0}=1$). By doing so, we can easily conclude that the probability of identification error in our problem using an ML decoder is upper bounded by

$$\begin{array}{cc} P[\;\mathrm{Identification}\;\mathrm{error}\; & \left|\;\mathrm{No}\;\mathrm{synchronization}\;\mathrm{error},\;\mathrm{No-collision}\;\right] \end{array}$$

(A24) $$\begin{array}{cc} \; & \leq \sum_{1\leq i<j\leq |W|}e^{-2nB\left(\left[P_{i}W_{i}\right],\left[P_{j}W_{j}\right]\right)} \end{array}$$

(A25) $$\begin{array}{cc} \; & \leq \sum_{1\leq i<j\leq |W|}e^{-2n\inf_{i,j}B\left(\left[P_{i}W_{i}\right],\left[P_{j}W_{j}\right]\right)} \end{array}$$

which goes to zero for |W|=poly(n) if $0<\inf_{i,j}B\left(\left[P_{i}W_{i}\right],\left[P_{j}W_{j}\right]\right)$. This corresponds to the fourth bound in (24).

**Decoding Step.** After finding the permutation of users in the active blocks, we can go ahead with the third stage of the achievable scheme to find the transmitted messages of the users using a maximum likelihood decoder. In this stage, we can group each set of users (with group size $N_{j},j\in \left[|W|\right]$ defined in (25)) who have similar channel $W_{j}$. In each block of each group we have to distinguish among $e^{R+\nu_{j}}$ messages and with similar steps as the proof of Theorem 1, we have

(A26) $$\begin{array}{cc} \; & P\left[\;\mathrm{Decoding}\;\mathrm{error}\;|\;\mathrm{No}\;\mathrm{synchronization},\;\mathrm{No}\;\mathrm{identification}\;\mathrm{error},\;\mathrm{No-collision}\right]\\ \; & \leq \sum_{j=1}^{\left|W\right|}P\left[\begin{array}{c} \;\mathrm{Decoding}\;\mathrm{error}\;\mathrm{for}\\ \;\mathrm{users}\;\mathrm{with}\;\mathrm{channel}\;W_{j} \end{array}|\;\mathrm{No}\;\mathrm{synchronization}\;\mathrm{and}\;\mathrm{identification}\;\mathrm{error},\;\mathrm{No-collision}\right]\\ \; & \leq \sum_{j=1}^{\left|W\right|}N_{j}e^{-nE_{r}\left(R+\nu_{j},P_{j},W_{j}\right)}=\sum_{j=1}^{\left|W\right|}e^{n\left(\nu_{j}-E_{r}\left(R+\nu_{j},P_{j},W_{j}\right)\right)}, \end{array}$$

which retrieves the second bound in (24). Finally, as for a synchronous channel and since we have to distinguish among $e^{R+\nu_{j}}$ messages in each block for some ϵ>0, we must have

(A27) $$\begin{array}{c} R+\nu_{j}+\epsilon <I\left(P_{j},W_{j}\right),\forall j\in \left[|W|\right], \end{array}$$

which corresponds to the last bound in (24).

Finally to conclude the proof, we should note that the bounds derived in (A23), (A26) and (A27) are all achievable bounds for each user for large enough block length *n* (say for $n\geq n_{0}$). Since we need the intersection of the achievable bounds for all users while the number of users itself increases with *n*, we need to express the asymptotic bound with the definition of *n* going to infinity. By expressing the achievable set for all users as $A_{n}$ defined in (24) by the equivalence of $\lim\inf_{n\to \infty }A_{n}\equiv {⋃}_{n_{0}=1}^{\infty }{⋂}_{n=n_{0}}^{\infty }A_{n}$ and by noting that for some $n_{0}$ every region $A_{n\geq n_{0}}$ is achievable by our achievability scheme previously introduced here, we see that (23) is indeed achievable.

**Remark** **A2.**
*Note that the existence of ϵ>0 in our bounds are due to the fact that we have a summation that depends on n and our exponent also decays exponentially with n while the n multiplier depends on the summation arguments. By including a constant ϵ>0 that multiplies n, we guarantee that the summation as a whole decays to zero.*

**Remark** **A3.**
*The achievability proof of Theorem 1 relies on constant composition codes whereas the achievability proof of Theorem 3 relies on i.i.d. codebooks. The reason for these restrictions is that in Theorem 3 we also need to distinguish different users and in order to use the result of [13], we focused our attention on i.i.d. codebooks.*

## Appendix G. Proof of Theorem 4

For the proof of Theorem 4, we use an ML decoder sequentially to perform the synchronization, identification and decoding all together, for each block.

**Proof.** *Codebook generation:* Each user $i\in [K_{n}]$ generates an i.i.d. random codebook according to the distribution $P_{i}$. *Probability of error analysis:* For each block $s\in [A_{n}]$, the decoder outputs

$$\begin{array}{c} i^{*}=\arg\max_{i\in \left[0:K_{n}\right],m\in \left[M_{n}\right]}Q_{i}\left(y_{s}^{n}|x_{i}^{n}\left(m\right)\right)\;, \end{array}$$

where $x_{0}^{n}=\emptyset$. We now find an upper bound on the probability of error given the hypothesis $H^{\left(1\right)}$ in (A4) for this decoder as follows

(A28a) $$\begin{array}{cc} \; & P_{e}^{\left(n\right)}\leq \sum_{i\in [K_{n}]}\sum_{m\in [2:M_{n}]}P\left[\log\frac{Q_{i}\left(Y_{i}^{n}|x_{i}^{n}\left(m\right)\right)}{Q_{i}\left(Y_{i}^{n}|x_{i}^{n}\left(1\right)\right)}>0|H^{\left(1\right)}\right] \end{array}$$

(A28b) $$\begin{array}{c} +\sum_{i\in [K_{n}]}\sum_{\begin{array}{c} j\in [0:K_{n}]\\ j\neq i \end{array}}\sum_{m\in [M_{n}]}P\left[\log\frac{Q_{j}\left(Y_{i}^{n}|x_{j}^{n}\left(m\right)\right)}{Q_{i}\left(Y_{i}^{n}|x_{i}^{n}\left(1\right)\right)}>0|H^{\left(1\right)}\right] \end{array}$$

(A28c)+∑s∈[Kn+1:An]∑j∈[Kn]∑m∈[Mn]PlogQj(Ysn|xjn(m))Q⋆(Ysn)>0|H(1)≤∑i∈[Kn]enRe−nsupt−logEQi(Yi∣X¯i)Qi(Yi∣Xi)t+∑i∈[Kn]∑j∈[0:Kn]j≠ienRe−nsupt−logEQj(Yi|Xj)Qi(Yi|Xi)t+enα∑j∈[Kn]enRe−nsupt−logEQj(Ys|Xj)Q⋆(Ys)t,

where $P_{X,\bar{X}}\left(x,x^{\prime }\right)=P_{X}\left(x\right)P_{X}\left(x^{\prime }\right)$. The term given in (A28a) is due to decoding error, (A28b) is due to identification error and finally (A28c) is due to synchronization error. The last inequality is by the Chernoff bound. In order for each term in the probability of error upper bound to vanish as *n* grows to infinity and by the explanation in the proof of Theorem 3 for the inclusion of n→∞ in our bounds, we find the conditions stated in the theorem. □

## Appendix H. Proof of (28)

To see (28a), we note that due to the symmetry in

$$C\left(P,Q,P,Q\right)=\underset{t}{\sup}-\log\sum_{x,x^{\prime },y}P\left(x\right)P\left(x^{\prime }\right)Q{\left(y|x\right)}^{1-t}Q{\left(y|x^{\prime }\right)}^{t},$$

the supremum is achieved at the midpoint $t=\frac{1}{2}$. hence $C\left(P,Q,P,Q\right)=\mu \left(\frac{1}{2}\right)$. Moreover, we find an upper bound on $C(\;.\;,Q_{⋆},P_{i},Q_{i})$ by noting that $\mu_{0,i}\left(t\right)$ defined in (12b) is concave in *t* with $\mu_{0,i}\left(1\right)=0$ and

$$\begin{array}{c} \frac{\partial \mu_{0,i}\left(t\right)}{\partial t}{|}_{t=1}=-I\left(P_{i},Q_{i}\right)-D\left(\left[P_{i}Q_{i}\right]\|Q_{⋆}\right)\leq 0. \end{array}$$

Hence $\mu_{0,i}\left(t\right)$ is always less than $\left(I\left(P_{i},Q_{i}\right)+D\left(\left[P_{i}Q_{i}\right]\|Q_{⋆}\right)\right)\left(1-t\right)$ and for 0≤t≤1 it is always less than $I\left(P_{i},Q_{i}\right)+D\left(\left[P_{i}Q_{i}\right]\|Q_{⋆}\right)$.

## Appendix I. Proof of Theorem 5

We first define the following special shorthand notations that we will use throughout this proof

(A29a) $$\begin{array}{cccc} F_{n} & :=M_{n}K_{n}, & Q_{i,m}^{n}\left(y^{n}\right) & :=Q_{i}\left(y^{n}|x_{i}^{n}\left(m\right)\right), \end{array}$$

(A29b) $$\begin{array}{cccc} P_{Y^{n}}\left(y^{n}\right) & :=\frac{1}{F_{n}}\sum_{i=1}^{K_{n}}\sum_{m=1}^{M_{n}}Q_{i,m}^{n}\left(y^{n}\right), & {Q_{i,m}^{n}}_{\lambda_{i}}\left(y^{n}\right) & :=\frac{{\left(Q_{i,m}^{n}\left(y^{n}\right)\right)}^{\lambda_{i}}{\left(Q_{⋆}^{n}\left(y^{n}\right)\right)}^{1-\lambda_{i}}}{\sum_{y^{n}}{\left(Q_{i,m}^{n}\left(y^{n}\right)\right)}^{\lambda_{i}}{\left(Q_{⋆}^{n}\left(y^{n}\right)\right)}^{1-\lambda_{i}}}, \end{array}$$

(A29c) $$\begin{array}{cccc} P_{Y^{n}}^{\left(\lambda \right)}\left(y^{n}\right) & :=\frac{1}{F_{n}}\sum_{i=1}^{K_{n}}\sum_{m=1}^{M_{n}}{Q_{i,m}^{n}}_{\lambda_{i}}\left(y^{n}\right), & {\left(P_{Y^{n}}\right)}_{t}\left(y^{n}\right) & :=\frac{{\left(P_{Y^{n}}\left(y^{n}\right)\right)}^{t}{\left(Q_{⋆}^{n}\left(y^{n}\right)\right)}^{1-t}}{\sum_{y^{n}}{\left(P_{Y^{n}}\left(y^{n}\right)\right)}^{t}{\left(Q_{⋆}^{n}\left(y^{n}\right)\right)}^{1-t}}, \end{array}$$

(A29d) $$\begin{array}{cc} Q_{⋆}^{n}\left(y^{n}\right):=\prod_{i=1}^{n}Q_{⋆}\left(y_{i}\right). & \end{array}$$

We use the optimal ML decoder to find the location of the ‘active’ blocks. In this stage, we are not concerned about the identity or the message of the users. In this regard, the decoder output is determined via

(A30) $$\begin{array}{c} \arg\max_{\begin{array}{c} \left(l_{1},\ldots ,l_{K_{n}}\right)\\ l_{i}\neq l_{j},\forall i\neq j\\ l_{i}\in \left[A_{n}\right],i\in \left[K_{n}\right] \end{array}}\sum_{i=1}^{K_{n}}\log\frac{\overset{-}{P_{Y^{n}}}\left(Y_{l_{i}}^{n}\right)}{Q_{⋆}^{n}\left(Y_{l_{i}}^{n}\right)}. \end{array}$$

Given the hypothesis that the users are active in blocks $\left[K_{n}\right]$, denoted by $H^{\left(1\right)}$ in (A4), the corresponding error events for the ML decoder are given by

(A31) $$\begin{array}{cc} \left\{\mathrm{error}|H^{\left(1\right)}\right\} & =\underset{\begin{array}{c} \left(l_{1},\ldots ,l_{K_{n}}\right)\\ \neq (1,\ldots ,K_{n}) \end{array}}{⋃}\left\{\sum_{i=1}^{K_{n}}\log\frac{P_{Y^{n}}\left(Y_{l_{i}}^{n}\right)}{Q_{⋆}^{n}\left(Y_{l_{i}}^{n}\right)}>\sum_{i=1}^{K_{n}}\log\frac{P_{Y^{n}}\left(Y_{i}^{n}\right)}{Q_{⋆}^{n}\left(Y_{i}^{n}\right)}\right\}\\ \; & ⊇\underset{\begin{array}{c} i\in [K_{n}]\\ j\in [K_{n}+1:A_{n}] \end{array}}{⋃}\left\{\log\frac{P_{Y^{n}}\left(Y_{j}^{n}\right)}{Q_{⋆}^{n}\left(Y_{j}^{n}\right)}\geq \log\frac{P_{Y^{n}}\left(Y_{i}^{n}\right)}{Q_{⋆}^{n}\left(Y_{i}^{n}\right)}\right\}\\ \; & ⊇\left\{\underset{\begin{array}{c} j\in [K_{n}+1:A_{n}] \end{array}}{⋃}\log\frac{P_{Y^{n}}\left(Y_{j}^{n}\right)}{Q_{⋆}^{n}\left(Y_{j}^{n}\right)}\geq T\right\}⋂\left\{\underset{i\in [K_{n}]}{⋃}T\geq \log\frac{P_{Y^{n}}\left(Y_{i}^{n}\right)}{Q_{⋆}^{n}\left(Y_{i}^{n}\right)}\right\}, \end{array}$$

for any T∈R. We take *T* to be

(A32) $$\begin{array}{c} T:=\frac{1}{F_{n}}\sum_{i=1}^{K_{n}}\sum_{m=1}^{M_{n}}\left(D\left({Q_{i,m}^{n}}_{\lambda_{i}}\|Q_{⋆}^{n}\right)-D\left({Q_{i,m}^{n}}_{\lambda_{i}}\|\overset{-}{P_{Y^{n}}}\right)\right), \end{array}$$

for different $\lambda_{i}\in \left[0,1\right],i\in \left[K_{n}\right]$.

We also find the following lower bounds

(A33a) $$\begin{array}{cc} Q_{⋆}^{n} & \left[\log\frac{\overset{-}{P_{Y^{n}}}}{Q_{⋆}^{n}}\left(Y^{n}\right)\geq T\right]\geq e^{-\frac{n}{K_{n}}\left(\sum_{i=1}^{K_{n}}D({Q_{i}}_{\lambda_{i}}\|Q_{⋆}\left|P_{i}\right)-\left(R+\nu \right)\left(1-{\overset{-}{r}}_{n}\right)+\frac{h({\overset{-}{r}}_{n})}{n}\right)}, \end{array}$$

(A33b) $$\begin{array}{cc} \overset{-}{P_{Y^{n}}} & \left[\log\frac{\overset{-}{P_{Y^{n}}}}{Q_{⋆}^{n}}\left(Y^{n}\right)\leq T\right]\geq e^{-\frac{n}{K_{n}}\sum_{i=1}^{K_{n}}D({Q_{i}}_{\lambda_{i}}\|Q_{i}\left|P_{i}\right)}, \end{array}$$

which are proved in Appendix K.

By using the inequalities in (A33a) and (A33b), we find a lower bound on the probability of (A31) as follows

(A34a) $$\begin{array}{cc} \; & P\left[\underset{\begin{array}{c} j\in [K_{n}+1A_{n}] \end{array}}{⋃}\log\frac{\overset{-}{P_{Y^{n}}}\left(Y_{j}^{n}\right)}{Q_{⋆}^{n}\left(Y_{j}^{n}\right)}\geq T\cap \underset{i\in [K_{n}]}{⋃}T\geq \log\frac{\overset{-}{P_{Y^{n}}}\left(Y_{i}^{n}\right)}{Q_{⋆}^{n}\left(Y_{i}^{n}\right)}|H^{\left(1\right)}\right] \end{array}$$

(A34b) $$\begin{array}{cc} \; & =P\left[\underset{\begin{array}{c} j\in [K_{n}+1:A_{n}] \end{array}}{⋃}\log\frac{\overset{-}{P_{Y^{n}}}\left(Y_{j}^{n}\right)}{Q_{⋆}^{n}\left(Y_{j}^{n}\right)}\geq T|H^{\left(1\right)}\right]P\left[\underset{i\in [K_{n}]}{⋃}T\geq \log\frac{\overset{-}{P_{Y^{n}}}\left(Y_{i}^{n}\right)}{Q_{⋆}^{n}\left(Y_{i}^{n}\right)}|H^{\left(1\right)}\right] \end{array}$$

(A34c) $$\begin{array}{cc} \; & =:P\left[Z_{1}\geq 1\right]P\left[Z_{2}\geq 1\right] \end{array}$$

(A34d) $$\begin{array}{cc} \; & \geq \left(1-\frac{\mathrm{Var}[Z_{1}]}{E^{2}\left[Z_{1}\right]}\right)\left(1-\frac{\mathrm{Var}[Z_{2}]}{E^{2}\left[Z_{2}\right]}\right) \end{array}$$

(A34e) $$\begin{array}{cc} \; & \geq \left(1-\frac{1}{\sum_{j=K_{n}+1}^{A_{n}}P\left[\xi_{j}=1\right]}\right)\left(1-\frac{1}{\sum_{i=1}^{K_{n}}P\left[\zeta_{i}=1\right]}\right) \end{array}$$

(A34f) $$\begin{array}{cc} \; & \geq \left(1-e^{-n\alpha +\frac{n}{K_{n}}\left(\sum_{i=1}^{K_{n}}D({Q_{i}}_{\lambda_{i}}\|Q_{⋆}\left|P_{i}\right)-\left(R+\nu \right)\left(1-{\overset{-}{r}}_{n}\right)+\frac{h({\overset{-}{r}}_{n})}{n}\right)}\right)\left(1-e^{-n\nu +\frac{n}{K_{n}}\sum_{i=1}^{K_{n}}D({Q_{i}}_{\lambda_{i}}\|Q_{i}\left|P_{i}\right)}\right), \end{array}$$

where (A34b) follows by independence of $Y_{i}^{n}$ and $Y_{j}^{n}$ whenever $i\neq j,\forall i,j\in [A_{n}]$ and the inequality in (A34d) is by Chebyshev’s inequality, where we have defined

(A35a) $$\begin{array}{cccc} Z_{1} & :=\sum_{j=K_{n}+1}^{A_{n}}\xi_{j}, & \; & \xi_{j}:=Ber\left(Q_{⋆}^{n}\left[\log\frac{\overset{-}{P_{Y^{n}}}}{Q_{⋆}^{n}}\left(Y_{j}^{n}\right)\geq T\right]\right), \end{array}$$

(A35b) $$\begin{array}{cccc} Z_{2} & :=\sum_{i=1}^{K_{n}}\zeta_{i}, & \; & \zeta_{i}:=Ber\left(\overset{-}{P_{Y^{n}}}\left[\log\frac{\overset{-}{P_{Y^{n}}}}{Q_{⋆}^{n}}\left(Y_{i}^{n}\right)\leq T\right]\right), \end{array}$$

where $\left\{\xi_{j},\zeta_{i}\right\}$ are mutually independent. We can see from (A34f) that if for some ϵ>0

(A36a) $$\begin{array}{cc} \nu & >\frac{1}{K_{n}}\sum_{i=1}^{K_{n}}D({Q_{i}}_{\lambda_{i}}\|Q_{i}\left|P_{i}\right)+\epsilon , \end{array}$$

(A36b) $$\begin{array}{cc} \alpha & >\frac{1}{K_{n}}\sum_{i=1}^{K_{n}}D({Q_{i}}_{\lambda_{i}}\|Q_{⋆}\left|P_{i}\right)-\left(1-{\bar{r}}_{n}\right)\left(\nu +R\right)+\epsilon , \end{array}$$

then the probability of error is strictly bounded away from zero and hence the region specified by (A36) (first bound in (31)) is impermissible.

We now focus only on decoding error probability, with the assumption that we have identified the active blocks. This is hence a lower bound on the overall probability of error. We have

(A37a) $$\begin{array}{cc} \; & P\left[\mathrm{Decoding}\;\mathrm{error}\;|\mathrm{No}\;\mathrm{synchronization}\;\mathrm{error}\right] \end{array}$$

(A37b) $$\begin{array}{cc} \; & =P\left[\overset{K_{n}}{\underset{i=1}{⋃}}\mathrm{Decoding}\;\mathrm{error}\;\mathrm{in}\;\mathrm{block}\;i\right] \end{array}$$

(A37c) $$\begin{array}{cc} \; & \geq 1-\frac{1}{\sum_{i=1}^{K_{n}}P\left[D_{i}^{c}\right]} \end{array}$$

(A37d) $$\begin{array}{cc} \; & \geq 1-\frac{1}{\sum_{i=1}^{K_{n}}e^{-nE_{sp}\left(R,P_{i},Q_{i}\right)}}, \end{array}$$

(A37e) $$\begin{array}{cc} \; & \geq 1-\frac{1}{K_{n}\;e^{-n\frac{1}{K_{n}}\sum_{i=1}^{K_{n}}E_{sp}\left(R,P_{i},Q_{i}\right)}}, \end{array}$$

(A37f) $$\begin{array}{cc} \; & =1-e^{-n\left(\nu -\frac{1}{K_{n}}\sum_{i=1}^{K_{n}}E_{sp}\left(R,P_{i},Q_{i}\right)\right)} \end{array}$$

where

$$D_{i}^{c}:=\;\mathrm{Decoding}\;\mathrm{error}\;\mathrm{in}\;\mathrm{block}\;i,$$

and where (A37a) can be derived using the same technique used in (A34). Finally, (A37d) is by lower bounding the probability of decoding error as in [14] where we defined $E_{sp}$ in (14). (A37e) is by Jensen’s inequality and finally as it can be seen in (A37f), if $\nu >\left(\frac{1}{K_{n}}\sum_{i=1}^{K_{n}}E_{sp}\left(R,P_{i},Q_{i}\right)\right)+\epsilon$ for an ϵ>0 the lower bound to the probability of decoding error is bounded away from zero for large values of *n* and this retrieves the second bound in (31). In addition, the usual converse bound on the rate of a synchronous channel also applies to any asynchronous channel and hence the region where R>I(P,Q)+ϵ is also impermissible. This concludes the proof.

## Appendix J. Proof of Theorem 6

User $i\in [K_{n}]$ has a codebook with $M_{n}=e^{nR}$ codewords of length *n*. Define for $i\in [K_{n}]$ an ‘extended codebook’ consisting of $A_{n}M_{n}$ codewords of length $nA_{n}$ constructed such that $\forall m\in [M_{n}]$ and $\forall t_{i}\in \left[A_{n}\right]$

$$\begin{array}{c} {\tilde{X}}_{i}^{nA_{n}}\left(m_{i},t_{i}\right):=\left[{⋆}_{i}^{n(t_{i}-1)}\;f_{i}\left(m_{i}\right)\;{⋆}_{i}^{n(A_{n}-t_{i})}\right], \end{array}$$

as depicted in Figure A4.

By using Fano’s inequality, i.e., $H\left(X_{1}^{nA_{n}},\ldots ,X_{K_{n}}^{nA_{n}}|Y^{nA_{n}}\right)\leq n\epsilon_{n}:\epsilon_{n}\to 0$ as n→∞, for any codebook of length $nA_{n}$ we have

$$\begin{array}{cc} \; & H\left(X_{1}^{nA_{n}},\ldots ,X_{K_{n}}^{nA_{n}}\right)=H\left(m_{1},t_{1},\ldots ,m_{K_{n}},t_{K_{n}}\right)\\ & =nK_{n}\left(\alpha +R\right)\\ & =H\left(X_{1}^{nA_{n}},\ldots ,X_{K_{n}}^{nA_{n}}|Y^{nA_{n}}\right)+I\left(X_{1}^{nA_{n}},\ldots ,X_{K_{n}}^{nA_{n}};Y^{nA_{n}}\right)\\ & \leq n\epsilon_{n}+ne^{n\alpha }\;\left|Y\right|\;⟺\\ & \nu +\frac{\log\left(1+\frac{1}{\alpha K_{n}}\sum_{i\in [K_{n}]}R_{i}\right)}{n}\leq \alpha +\frac{\log\left(1+\frac{\epsilon_{n}}{e^{n\alpha }|Y|}\right)}{n}, \end{array}$$

where $\frac{\log\left(1+\frac{1}{\alpha K_{n}}\sum_{i\in [K_{n}]}R_{i}\right)}{n}\geq 0$ and $\frac{\log(1+\frac{\epsilon_{n}}{e^{n\alpha }|Y|})}{n}\geq 0$ vanish as *n* goes to infinity.

## Appendix K. Proof of (A33a) and (A33b)

Before deriving lower bounds on (A33a) and (A33b), we note that by the Type-counting Lemma [19], at the expense of a small decrease in the rate (which vanishes in the limit for large blocklength) we may restrict our attention to constant composition codewords. Henceforth, we assume that the composition of the codewords for user $i\in [K_{n}]$ is given by $P_{i}$. Moreover, to make this paper self-contained, we restate the following Lemmas that we use in the rest of the proof.

**Lemma** **A2** (Compensation Identity). *For arbitrary $\pi_{i}:\sum_{i=1}^{K}\pi_{i}=1$ and arbitrary probability distribution functions $P_{i}\in P_{X},i\in \left[K\right]$, we define $\bar{P}\left(x\right)=\sum_{i=1}^{K}\pi_{i}P_{i}\left(x\right)$. Then for any probability distribution function R we have*

(A38) $$\begin{array}{cc} \; & D\left(\bar{P}\|R\right)+\sum_{i=1}^{K}\pi_{i}D\left(P_{i}\|\bar{P}\right)=\sum_{i=1}^{K}\pi_{i}D(P_{i}||R). \end{array}$$

**Lemma** **A3** (Fano). *Let F be an arbitrary set of size N. For $\bar{P}=\frac{\sum_{\theta \in F}P_{\theta }}{N}$ we have*

(A39) $$\begin{array}{c} \frac{1}{N}\sum_{\theta \in F}D\left(P_{\theta }\|\bar{P}\right)\geq \left(1-\bar{r}\right)\log\left(N(1-\bar{r})\right)+\bar{r}\log\left(\frac{N\bar{r}}{N-1}\right), \end{array}$$

*where*

(A40) $$\begin{array}{c} \bar{r}:=\underset{T}{\inf}\frac{1}{N}\sum_{\theta \in F}P_{\theta }\left\{T\neq \theta \right\} \end{array}$$

*in which the infimum is taken over all possible estimators T.*

We now continue with the proof of (A33a). Using the Chernoff bound we can write

(A41) $$\begin{array}{cc} Q_{⋆}^{n} & \left[\log\frac{P_{Y^{n}}}{Q_{⋆}^{n}}\left(Y^{n}\right)\geq \frac{1}{F_{n}}\sum_{i=1}^{K_{n}}\sum_{m=1}^{M_{n}}D\left({Q_{i,m}^{n}}_{\lambda_{i}}\|Q_{⋆}^{n}\right)-D\left({Q_{i,m}^{n}}_{\lambda_{i}}\|\overset{-}{P_{Y^{n}}}\right)\right]\overset{\mathrm{Chernoff}}{≐}e^{-\sup_{t}A\left(t\right)}. \end{array}$$

The Chernoff bound exponent, $\sup_{t}A\left(t\right)$, is expressed and simplified as follows

(A42) $$\begin{array}{cc} A(t) & :=\frac{t}{F_{n}}\sum_{i=1}^{K_{n}}\sum_{m=1}^{M_{n}}D\left({Q_{i,m}^{n}}_{\lambda_{i}}\|Q_{⋆}^{n}\right)-D\left({Q_{i,m}^{n}}_{\lambda_{i}}\|\overset{-}{P_{Y^{n}}}\right)-\log E_{Q_{⋆}^{n}}\left[{\left(\frac{\overset{-}{P_{Y^{n}}}}{Q_{⋆}^{n}}\left(Y^{n}\right)\right)}^{t}\right]\\ \; & =\frac{t}{F_{n}}\sum_{i=1}^{K_{n}}\sum_{m=1}^{M_{n}}\sum_{y^{n}}{Q_{i,m}^{n}}_{\lambda_{i}}\left(y^{n}\right)\log\frac{\overset{-}{P_{Y^{n}}}\left(y^{n}\right)}{Q_{⋆}^{n}\left(y^{n}\right)}-\log\sum_{y^{n}}{\left(\overset{-}{P_{Y^{n}}}\left(y^{n}\right)\right)}^{t}{\left(Q_{⋆}^{n}\left(y^{n}\right)\right)}^{1-t}\\ \; & =\frac{1}{F_{n}}\sum_{i=1}^{K_{n}}\sum_{m=1}^{M_{n}}\sum_{y^{n}}{Q_{i,m}^{n}}_{\lambda_{i}}\left(y^{n}\right)\log\frac{\frac{{\left(\overset{-}{P_{Y^{n}}}\left(y^{n}\right)\right)}^{t}{\left(Q_{⋆}^{n}\left(y^{n}\right)\right)}^{1-t}}{\sum_{y^{n}}{\left(\overset{-}{P_{Y^{n}}}\left(y^{n}\right)\right)}^{t}{\left(Q_{⋆}^{n}\left(y^{n}\right)\right)}^{1-t}}}{Q_{⋆}^{n}\left(y^{n}\right)}\\ \; & =\frac{1}{F_{n}}\sum_{i=1}^{K_{n}}\sum_{m=1}^{M_{n}}\sum_{y^{n}}{Q_{i,m}^{n}}_{\lambda_{i}}\left(y^{n}\right)\log\frac{{\left(\overset{-}{P_{Y^{n}}}\right)}_{t}\left(y^{n}\right)}{Q_{⋆}^{n}\left(y^{n}\right)}\\ \; & =\frac{1}{F_{n}}\sum_{i=1}^{K_{n}}\sum_{m=1}^{M_{n}}D\left({Q_{i,m}^{n}}_{\lambda_{i}}\|Q_{⋆}^{n}\right)-D\left({Q_{i,m}^{n}}_{\lambda_{i}}\|{\left(\overset{-}{P_{Y^{n}}}\right)}_{t}\right)\\ \; & =\frac{1}{K_{n}}\sum_{i=1}^{K_{n}}nD\left({Q_{i}}_{\lambda_{i}}\left\|Q_{⋆}\right|P_{i}\right)-\frac{1}{F_{n}}\sum_{i=1}^{K_{n}}\sum_{m=1}^{M_{n}}D\left({Q_{i,m}^{n}}_{\lambda_{i}}\|{\left(\overset{-}{P_{Y^{n}}}\right)}_{t}\right), \end{array}$$

where (A42) is the result of the constant composition structure of the codewords. As a result,

(A43) $$\begin{array}{c} \underset{t}{\sup}A\left(t\right)=\frac{1}{K_{n}}\sum_{i=1}^{K_{n}}nD\left({Q_{i}}_{\lambda_{i}}\left\|Q_{⋆}\right|P_{i}\right)-\underset{t}{\inf}\left\{\frac{1}{F_{n}}\sum_{i=1}^{K_{n}}\sum_{m=1}^{M_{n}}D\left({Q_{i,m}^{n}}_{\lambda_{i}}\|{\left(\overset{-}{P_{Y^{n}}}\right)}_{t}\right)\right\}. \end{array}$$

Moreover,

$$\begin{array}{cc} \; & \underset{t}{\inf}\frac{1}{F_{n}}\sum_{i=1}^{K_{n}}\sum_{m=1}^{M_{n}}D\left({Q_{i,m}^{n}}_{\lambda_{i}}\|{\left(\overset{-}{P_{Y^{n}}}\right)}_{t}\right)\\ \; & =\underset{t}{\inf}\frac{1}{F_{n}}\sum_{i=1}^{K_{n}}\sum_{m=1}^{M_{n}}\sum_{y^{n}}{Q_{i,m}^{n}}_{\lambda_{i}}\left(y^{n}\right)\log\frac{{Q_{i,m}^{n}}_{\lambda_{i}}\left(y^{n}\right)}{{\left(\overset{-}{P_{Y^{n}}}\right)}_{t}\left(y^{n}\right)}\\ \; & =\underset{t}{\inf}\frac{1}{F_{n}}\sum_{i=1}^{K_{n}}\sum_{m=1}^{M_{n}}\sum_{y^{n}}{Q_{i,m}^{n}}_{\lambda_{i}}\left(y^{n}\right)\log\frac{{Q_{i,m}^{n}}_{\lambda_{i}}\left(y^{n}\right)}{{\left(\overset{-}{P_{Y^{n}}}\right)}_{t}\left(y^{n}\right)}\cdot \frac{\overset{-}{P_{Y^{n}}^{\left(\lambda \right)}}\left(y^{n}\right)}{\overset{-}{P_{Y^{n}}^{\left(\lambda \right)}}\left(y^{n}\right)}\\ \; & =\underset{t}{\inf}\frac{1}{F_{n}}\sum_{i=1}^{K_{n}}\sum_{m=1}^{M_{n}}D\left({Q_{i,m}^{n}}_{\lambda_{i}}\|\overset{-}{P_{Y^{n}}^{\left(\lambda \right)}}\right)+D\left(\overset{-}{P_{Y^{n}}^{\left(\lambda \right)}}\|{\left(\overset{-}{P_{Y^{n}}}\right)}_{t}\right)\\ \; & \geq \frac{1}{F_{n}}\sum_{i=1}^{K_{n}}\sum_{m=1}^{M_{n}}D\left({Q_{i,m}^{n}}_{\lambda_{i}}\|\overset{-}{P_{Y^{n}}^{\left(\lambda \right)}}\right). \end{array}$$

Note that $\overset{-}{P_{Y^{n}}^{\left(\lambda \right)}}$ is the average of ${Q_{i,m}^{n}}_{\lambda_{i}}$ over m,i’s ($m\in \left[M_{n}\right],i\in \left[K_{n}\right]$) and hence based on Lemma A3, we have

$$\begin{array}{cc} \frac{1}{F_{n}}\sum_{i=1}^{K_{n}}\sum_{m=1}^{M_{n}}D\left(Q_{i,m}^{n}\|\overset{-}{P_{Y^{n}}^{\left(\lambda \right)}}\right) & \geq \left(1-{\bar{r}}_{n}\right)\log\left(F_{n}\left(1-{\bar{r}}_{n}\right)\right)+{\bar{r}}_{n}\log\left(\frac{F_{n}{\bar{r}}_{n}}{F_{n}-1}\right)\\ & \geq \left(1-{\bar{r}}_{n}\right)\log F_{n}-h\left({\bar{r}}_{n}\right). \end{array}$$

As a result

$$\begin{array}{cc} \underset{t}{\sup}A\left(t\right) & \leq \frac{1}{K_{n}}\sum_{i=1}^{K_{n}}nD({Q_{i}}_{\lambda_{i}}\|Q_{⋆}\left|P_{i}\right)-n\left(R+\nu \right)\left(1-{\bar{r}}_{n}\right)+h\left({\bar{r}}_{n}\right). \end{array}$$

Now we continue with the proof of (A33b). Again, using the Chernoff bound we have

$$\begin{array}{cc} \; & \overset{-}{P_{Y^{n}}}\left[\log\frac{P_{Y^{n}}}{Q_{⋆}^{n}}\left(Y^{n}\right)\leq \frac{1}{F_{n}}\sum_{i=1}^{K_{n}}\sum_{m=1}^{M_{n}}D\left({Q_{i,m}^{n}}_{\lambda_{i}}\|Q_{⋆}^{n}\right)-D\left({Q_{i,m}^{n}}_{\lambda_{i}}\|\overset{-}{P_{Y^{n}}}\right)\right]\\ \; & =\overset{-}{P_{Y^{n}}}\left[\log\frac{Q_{⋆}^{n}}{P_{Y^{n}}}\left(Y^{n}\right)\geq \frac{1}{F_{n}}\sum_{i=1}^{K_{n}}\sum_{m=1}^{M_{n}}D\left({Q_{i,m}^{n}}_{\lambda_{i}}\|\overset{-}{P_{Y^{n}}}\right)-D\left({Q_{i,m}^{n}}_{\lambda_{i}}\|Q_{⋆}^{n}\right)\right]≐e^{-\sup_{t}B\left(t\right)}, \end{array}$$

where

(A44) $$\begin{array}{cc} \underset{t}{\sup}B\left(t\right) & :=\underset{t}{\sup}\frac{t}{F_{n}}\sum_{i=1}^{K_{n}}\sum_{m=1}^{M_{n}}\sum_{y^{n}}{Q_{i,m}^{n}}_{\lambda_{i}}\left(y^{n}\right)\log\frac{Q_{⋆}^{n}\left(y^{n}\right)\overset{-}{P_{Y^{n}}}\left(y^{n}\right)}{\overset{-}{P_{Y^{n}}}\left(y^{n}\right)}-\log\sum_{y^{n}}{\left(Q_{⋆}^{n}\left(y^{n}\right)\right)}^{t}{\left(\overset{-}{P_{Y^{n}}}\left(y^{n}\right)\right)}^{1-t}\\ \; & =\underset{t}{\sup}\sum_{y^{n}}\overset{-}{P_{Y^{n}}^{\left(\lambda \right)}}\left(y^{n}\right)\log\frac{{\left(\overset{-}{P_{Y^{n}}}\right)}_{1-t}\left(y^{n}\right)}{\overset{-}{P_{Y^{n}}}\left(y^{n}\right)}\\ \; & =\underset{t}{\sup}D\left(\overset{-}{P_{Y^{n}}^{\left(\lambda \right)}}\|\overset{-}{P_{Y^{n}}}\right)-D\left(\overset{-}{P_{Y^{n}}^{\left(\lambda \right)}}\|{\left(\overset{-}{P_{Y^{n}}}\right)}_{1-t}\right)\\ \; & \leq D\left(\overset{-}{P_{Y^{n}}^{\left(\lambda \right)}}\|\overset{-}{P_{Y^{n}}}\right)=\sum_{y^{n}}\left(\frac{1}{F_{n}}\sum_{i=1}^{K_{n}}\sum_{m=1}^{M_{n}}{Q_{i,m}^{n}}_{\lambda_{i}}\left(y^{n}\right)\right)\log\left(\frac{\frac{1}{F_{n}}\sum_{i=1}^{K_{n}}\sum_{m=1}^{M_{n}}{Q_{i,m}^{n}}_{\lambda_{i}}\left(y^{n}\right)}{\frac{1}{F_{n}}\sum_{i=1}^{K_{n}}\sum_{m=1}^{M_{n}}Q_{i,m}^{n}\left(y^{n}\right)}\right)\\ \; & \leq \sum_{y^{n}}\frac{1}{F_{n}}\sum_{i=1}^{K_{n}}\sum_{m=1}^{M_{n}}{Q_{i,m}^{n}}_{\lambda_{i}}\left(y^{n}\right)\log\frac{{Q_{i,m}^{n}}_{\lambda_{i}}\left(y^{n}\right)}{Q_{i,m}^{n}\left(y^{n}\right)}=\frac{1}{F_{n}}\sum_{i=1}^{K_{n}}\sum_{m=1}^{M_{n}}D\left({Q_{i,m}^{n}}_{\lambda_{i}}\|Q_{i,m}^{n}\right)\\ \; & =\frac{1}{K_{n}}\sum_{i=1}^{K_{n}}nD\left({Q_{i}}_{\lambda_{i}}\left\|Q\right|P_{i}\right), \end{array}$$

and where the inequality in (A44) is by the Log-Sum inequality.

## Appendix L. Proof of Lemma 1

We will prove the Lemma by contradiction. Define

$$t_{i}≜\mathrm{Number}\;\mathrm{of}\;\mathrm{users}\;\mathrm{in}\;\mathrm{block}\;\;i.$$

Assume that the arrangement with highest probability (let us call it A) has at least two blocks, say blocks 1,2, for which $t_{1}-t_{2}>1$. This assumption means that the arrangement with the highest probability is *not* the non-overlapping arrangement.

The probability of this arrangement, P(A), is proportional to

P(A)∝Knt1Kn−t1t2=Kn!t1,(Kn−t1)!(Kn−t1)!t2!(Kn−t1−t2)!=Kn!t1!t2!(Kn−t1−t2)!.

We now consider a new arrangement, $A_{\mathrm{new}}$, in which $t_{1,\mathrm{new}}=t_{1}-1$ and $t_{2,\mathrm{new}}=t_{2}+1$ and all other blocks remain unchanged. This new arrangement is also feasible since we have not changed the number of users. The probability of this new arrangement is proportional to

P(Anew)∝Knt1−1Kn−t1−1t2+1=Kn!(t1−1)!(Kn−t1+1)!(Kn−t1+1)!(t2+1)!(Kn−t1−t2)!=Kn!(t1−1)!(t2+1)!(Kn−t1−t2)!.

Comparing P(A) and $P(A_{\mathrm{new}})$ we see that $P\left(A\right)<P\left(A_{\mathrm{new}}\right)$ which is a contradiction to our primary assumption that A has the highest probability among all arrangements. Hence there do not exist two blocks which differ more than one in the number of active users within them in the arrangement with the highest probability.
